# Understanding the child and adolescent eating disorder treatment experiences of autistic people and parents

**DOI:** 10.1186/s40337-025-01331-w

**Published:** 2025-07-06

**Authors:** Colleen Alford, Andrew Wallis, Phillipa Hay, Deborah Mitchison

**Affiliations:** 1 Sydney Children’s Hospitals Network, Eating Disorders Service, Department of Psychological Medicine, Locked Bag 4001, Westmead, New South Wales, 2145, Sydney, Australia; 2 Graduate School of Health, Faculty of Health, Discipline of Psychology, University of Technology Sydney, Sydney, Australia; 3 Translational Health Research Institute, Western Sydney University, Sydney, Australia; 4 Mental Health Services, South Western Sydney Local Health District, Campbelltown, New South Wales Australia

**Keywords:** Autism, Eating disorder, Treatment experiences, Children, Adolescents, Parents

## Abstract

**Background:**

Autistic people are overrepresented in eating disorders services and often require more intensive and extended treatment. Little is known about the eating disorder treatment experiences of autistic young people. The aim of this research was to understand the child and adolescent eating disorder treatment experiences of autistic people and parents of autistic young people.

**Methods:**

Constructivist grounded theory was employed. Nine autistic people with lived experience of an eating disorder as an adolescent as well as nine parents of autistic young people engaged in paediatric eating disorders treatment were recruited through purposive sampling and then interviewed using a semi-structured design. Each interview was transcribed and analysed to identify themes and develop emergent theory directly from the data.

**Results:**

Two major themes emerged (1) Misunderstood and (2) Safe and supportive eating disorder treatment for autistic young people and their families. Being ‘Misunderstood’ was found to occur across eight different domains including problems related to diagnosis, misattribution, neuro-normative definitions of eating and recovery, a one size fits all approach, siloed expertise, limited expertise and treatment options for eating disorders outside of anorexia nervosa, family neurodivergence and autism not being accommodated. The effects of being misunderstood included an increased burden of case management and advocacy, distress and trauma, mistrust of health professionals, identity disruption, and setbacks in recovery. ‘Safe and supportive eating disorders treatment for autistic young people and their families’ is informed by lived experience and built on a foundation of general care principles and autism-specific elements.

**Conclusions:**

Being misunderstood is a recursive process that occurs across multiple aspects of eating disorder care. Autistic young people and their families desire and need safety and understanding. This is achieved through care characteristics that promote connection and nuance, and autism specific adaptations and accommodations that provide optimal support conditions and strengthen therapeutic alliance.

**Trial registration:**

This research was approved for prospective registration on Open Science Framework (OSF) on the 8th December 2023.

**Supplementary Information:**

The online version contains supplementary material available at 10.1186/s40337-025-01331-w.

## Abstract (plain language summary)

We know very little about the eating disorder treatment experiences of autistic young people under the age of eighteen. This study therefore sought to understand the child and adolescent eating disorder treatment experiences of autistic people and parents of autistic people through semi-structured interviews. Being misunderstood across different aspects of eating disorders care was a common experience resulting in a range of detrimental consequences. We found that there are some key treatment principles that promote safety and understanding for autistic young people and their families, and only on the basis of this safety and understanding can eating disorders treatment be effective and facilitate change.

**Supplementary Information:**

The online version contains supplementary material available at 10.1186/s40337-025-01331-w.

## Background

Eating disorders have a significant impact on children and adolescents and their families. One in five children or adolescents experience an eating disorder [1] and up to 22% of children and young people with eating disorders are autistic [2]^1^ or have elevated autistic traits [3]. This figure is much higher than community prevalence of autism which sits at an estimated 1% of the population [4, 5].

While it is acknowledged that autistic people are over-represented in eating disorder populations [6, 7] there remains much to be understood about the interplay between autism and eating disorders [8]. Of particular importance is autistic people’s experience of eating disorder treatment and how it needs to be adapted.

Arguments in favour of adapting standard eating disorders care for autistic people are based on the poorer eating disorder treatment outcomes documented in treatment studies, as well as lived-experience accounts calling specifically for adaptations. In a recent rapid review of eating disorder treatment outcomes of 1320 studies, Miskovic-Wheatley and colleagues [9] identified that a shorter duration of illness was associated with more positive treatment outcomes, emphasising the importance of timely access to effective treatment and identifying any barriers to such. The rapid review by Miskovic-Wheatley and colleagues also highlighted that neurodivergence was one of the factors associated with poorer treatment outcomes [9], again adding to the urgent need to understand how best to improve eating disorders treatment outcomes for autistic people. While eating disorder treatment completion rates and weight-based outcomes are similar for autistic people compared to non-autistic people, autistic people often require more intensive eating disorder treatment including increased inpatient medical or psychiatric admission days [10–12], increased involvement in day programs as opposed to outpatient care [10, 12], and nasogastric tube use [11]. In fact, one study found a direct correlation between number of autistic traits (as measured by the DSM-5 criteria identified in chart notes) and number of days in eating disorder treatment [13]. Some studies have also demonstrated poor eating disorder treatment outcomes when considering broader social-emotional and socio-economic outcomes [14–16]. Only one study of a paediatric cohort in Italy, found no difference in eating disorder treatment outcomes including treatment intensity [17].

Studies that have sought the perspectives of autistic people with lived experience of eating disorders have consistently found that autistic people do not feel understood, and that treatment is not adapted or tailored to their needs [18–20]. Carers and supporters of autistic people with lived experience of eating disorders also spoke of encountering a lack of knowledge about autism, and the need for autism specific accommodations and personalised care [19, 21]. These lived experience accounts provide compelling reasons for eating disorder services to be improved. It is important to note that these studies focus on the treatment experiences of autistic adults. There is a lack of research on the eating disorder treatment experiences of autistic young people (aged < 18 years) and their parents/carers. In the recent systematic review on the experiences of autistic people with restrictive eating disorders, Loomes and colleagues [20] identified, in the absence of existing studies, the pressing need for research that seeks to understand the lived experience perspectives of autistic young people with restrictive eating disorders. This is an important gap to address given that the peak age of onset of eating disorders is middle adolescence and many people with eating disorders first experience eating disorder treatment during their teen years [22].

Eating disorder clinicians have expressed a lack of confidence in working with autistic people with eating disorders and any implementation of adaptations seem to rely on individual clinician experience [23]. A recent study of clinician experiences of eating disorder focused family therapy with autistic young people found that while some clinicians are making autism-specific treatment adaptations there is a dilemma around retaining fidelity of empirically-supported treatments while making autism-related accommodations or adaptations [24]. This lack of confidence and consistency highlights the need for clinicians to learn from lived experience perspectives about how treatment needs to be adapted for autistic young people to feel safe and supported. The value of lived experience accounts informing treatment cannot be overstated.

Despite the identified need, there are very limited published treatment adaptations for autistic people with eating disorders. In Li and colleagues’ systematic review of treatment adaptations only three studies outlining treatment adaptations were identified and these were all published by the same research group, the Pathway for Eating Disorders and Autism developed from Clinical Experience (PEACE Pathway) [25]. The PEACE Pathway is a comprehensive, multidisciplinary approach to tailoring and improving eating disorder treatment for autistic people [26, 27]. It has been recently adapted for children and adolescents with promising results in regards to child and adolescent mental health outcomes as well as clinician confidence [28]. Some clinical recommendations for adapting Family Based Treatment for Anorexia Nervosa for autistic young people have also been published [29].

While some treatment recommendations are emerging for autistic children and adolescents with eating disorders [28, 29] there are yet to be any published studies exploring the perspectives of autistic people about their eating disorders treatment as a young person specifically. Autistic and lived experience accounts are critical to informing clinical practice and guidelines [30, 31] and this acts as a counter to marginalisation and pathologisation of autistic people.

Given the high heritability of autism and the importance of parent involvement in paediatric eating disorders treatment, the perspectives of autistic parents are also essential to consider. Researchers have been interested in the prevalence of autism in parents of people with eating disorders [32, 33] but to our knowledge there are no qualitative studies that examine the eating disorder treatment experience of autistic parents.

Therefore the first aim of this study was to explore the experiences of autistic people regarding eating disorders treatment during adolescence. The perspectives of parents and carers of autistic young people with eating disorders were also sought given the important role that parents play in child and adolescent eating disorders treatment [34, 35]. The treatment experiences of autistic and non-autistic parents were compared to determine the specific experiences and needs of autistic parents.

The second aim of this study was to ascertain what autistic people and their parents need and want from paediatric eating disorders treatment so that the autistic voice can be privileged in considering implications for clinical practice and future directions for eating disorders treatment research. In recent studies of autistic people’s priorities for research, mental health and accessible health care were key priorities [36, 37]. Research that focused on applied research (research that has clinical or day-to-day implications) has also been highlighted as a priority by the autistic community [38]. The aims of this study are in alignment with the se priorities.

## Methods

### Design

The initial idea for this study came from a focus group of parents of autistic young people with eating disorders that was conducted as part of a quality improvement project (2016–2025) in a specialist paediatric eating disorders service in Australia.

This study utilised Constructivist Grounded Theory [39] methodology to understand the eating disorder treatment experiences of autistic young people and parents. Constructivist Grounded Theory methodology [40] was chosen for two key reasons, firstly the dearth of literature on the eating disorder treatment experiences of autistic young people necessitated the development of theory. The aim of the grounded theory approach is to generate theories rather than focusing on proving or disproving existing theories [41] thus the aims of grounded theory research align with the aims of this research. Secondly, constructivist grounded theory was undertaken in recognition that the researcher is not removed from the research process and theory development but brings their own personal and professional experiences, knowledge, and interactional processes [42].

The research has been reported using the Consolidated Criteria for Reporting Qualitative Research (COREQ) [43].

### Reflexivity

A reflexive stance is critical to the constructivist process [44] and multiple process were in place to promote reflexivity including fortnightly research supervision, personal reflective writing, and meeting with lived experience consultants and prominent experts in this field to discuss inclusivity, neuro-affirming research and treatment, and general research processes.

The reflexivity statement of the primary author is below, and the reflexivity statements from the other authors can be found at the end of the manuscript.

Colleen Alford: I am a white cisfemale PhD candidate at the University of Technology Sydney and social worker in a specialist public eating disorders service. My research and clinical work focuses on eating disorders treatment and therapy for children, adolescents, and their families. I have worked in the eating disorders field for eighteen years. My interest in autism and eating disorders grew out of working with many autistic young people experiencing eating disorders. I have valued the way that these young people and their families have provided feedback and shared their experiences.

### Ethical considerations

All participants received written and verbal information about the study and provided written informed consent. This study was approved by the Sydney Children’s Hospitals Network Human Research Ethics Committee (2023/ETH01336) and this was ratified by the University of Technology Sydney Research Ethics Committee (ETH23-8864).

### Recruitment

Participants were eligible to participate in the study if they were either a parent of an autistic young person (18 or under) with an eating disorder, or an autistic person with lived experience of an eating disorder and eating disorder treatment as a child or adolescent. If participants were currently over 18 years of age, they needed to be able to reflect on their adolescent experiences. Any data provided relating to their adult eating disorder treatment was not analysed as part of this study.

The scope of the study was as broad as possible to understand multiple perspectives. All eating disorder diagnoses were therefore included, and there were no criteria placed on what type of eating disorders treatment autistic young people had experienced. Other eligibility criteria included an autism diagnosis (self or child) made by a health professional, ability to complete a research interview, and aged 16 years or over.

Participants were recruited through social media, clinicians and services in the researchers’ networks, and referrals from other participants.

### Participants

In line with grounded theory methodology, purposive sampling was utilised [45]. Prospective participants accessed study information and the study eligibility screener either through a QR code link or by emailing the researcher directly. The initial screening questions and consent were completed by 54 individuals. Of these, 36 were deemed ineligible or were not contactable when an interview time was offered. The remaining 18 participants went on to attend an interview with the first author and were included in this study. Nine of these participants were autistic people reflecting on their treatment experiences as an adolescent, and nine of the participants were parents of autistic young people reflecting on their experience of treatment from a carer perspective. Any parent participants who identified as autistic with lived experience of an eating disorder as an adolescent were classified as parent participants because most of the experiences they discussed were experiences related to their children.

Table 1, seen below, summarises the demographic data of participants..

**Table 1:** A table summarising the demographic information of participants

	Parents	Young People
Age (years), M, SD, range	Mean: 48.1 SD: 7.04 Range: 33–55	Mean: 22.9 SD: 5.96 Range: 16–36*
Gender (n)		
Girl/woman	8	6
Boy/man	1	0
Non-binary	0	3
Location (n)		
NSW	2	4
VIC	3	1
WA	2	0
SA	2	1
ACT	0	2
QLD	0	1
Urbanicity		
Metropolitan (n)	1	6
Rural/Regional (n)	8	3
Eating disorder diagnosis (self or of child) (n)		
Anorexia nervosa	4	5
ARFID	3	0
Binge eating disorder	2	0
EDNOS	1	0
Mixed eating disorder presentation	0	4
Age on eating disorder onset (M, SD, range)**	M: 8.45 R: 1–14 SD: 5.96	M: 14 R: 11–17 SD: 4.17
Type of child and adol eating disorders treatment (no. of yp)		
Inpatient only	0	0
Outpatient only	5	5
Both inpatient and outpatient	5	4
Age of autism diagnosis (M, SD, range)	M: 11.09 R: 2–17 SD: 4.58	M:19.88 R:12–35 SD: 6.59
Timing of autism diagnosis (no. of yp)		
Autism < ED treatment	6	1
During child/adolescent ED treatment	4	3
As an adult	0	5
Parent neurodivergence		
Not autistic	4	
Formally diagnosed	2	
Self-diagnosed	2	
Possibly autistic	1	

*N* = 18 participants; 18 people’s child and adolescent eating disorders explored (2 parents had 2x autistic children with eating disorders to discuss; 2 parent participants discussed their same child; 1 autistic young person and 1 parent were from the same family)

*Age of autistic participants: 16, 17, 20, 21, 22, 23, 24, 27, 36

**Age of onset of eating disorder was self-reported and varied between eating disorder diagnoses, with age of onset of ARFID symptoms being the lowest (m:2.6 years of age)

All participant names and identifying information (treatment providers, locations, dates) were removed from transcripts and selected quotes in this manuscript and replaced with pseudonyms to retain confidentiality.

### Data collection materials

#### Screening and demographic questionnaire

The screening and demographic questionnaire asked questions around age of eating disorder onset, and details of the autism diagnosis to ensure eligibility. Questions relating to age, postcode, type of eating disorder, age of autism diagnosis were also included to capture some initial demographic information. Gender and ethnicity questions were asked as part of the research interview.

#### Interview outline

The interview outline (see supplementary material) served as a guide for the interview and was provided to participants prior to the interview. The interview questions focused on the type of eating disorders treatment the participant/participant’s child had received, overall treatment experience, helpful and unhelpful aspects of treatment, and suggestions for improving eating disorders treatment for autistic young people.

The interview outline was reviewed by a board member of Eating Disorders Neurodiversity Australia, a not-for-profit neurodivergent-led organisation. As per the guidance received, the interview questions were modified and participants were given options of how they wished to communicate including whether to have their video on, or to use the chat function rather than speaking.

### Procedure

All participants completed a screening and demographic questionnaire. The researcher contacted each prospective participant to provide further information and plan the research interview and discuss accommodations that were needed to make the interview accessible and safe. Participants were given the choice of an in-person interview or an online interview. All but one participant chose an online interview.

Whereas generally interviews were scheduled one-on-one with participants, one interview was conducted with two participants (a couple) who requested a joint interview. Two participants were mother and daughter but were interviewed separately. Two participants discussed their experiences of multiple autistic children with eating disorders. Interviews took 40–120 min (average 70 min). The semi-structured interviews were conducted by the first author using the interview outline. Questions outside of the interview outline were asked in direct response to participant experiences to ensure depth of understanding. As part of the iterative interview process further questions about the experience of therapy were asked in the latter interviews as it was identified that while data on hospital experiences had reached saturation, a greater sense of outpatient therapy experiences were needed to continue to develop emerging themes.

Interviews were recorded using Zoom™. The automatic transcription features were utilised, and transcripts were read and edited soon after the interview concluded, aided when needed by reference to the video recording. All participants were asked if they would like to receive a copy of their transcribed interview and were invited to advise of any changes. Five participants requested their transcript and no requests for corrections were received. Participants were also invited to provide further information after the interview and additional reflections and information were received via email from six participants. These additions were included with their transcripts for analysis.

### Data analysis

In line with Grounded Theory methodology, data collection and analysis occurred simultaneously [41]. Memoing was used during the interviews and immediately after interviews to capture initial ideas and reflections and prompt further exploration [39]. De-identified verbatim transcripts were uploaded to NVivo (Version 14.0). Open coding was used initially to develop codes followed by focused coding which explores meaning and connections [39]. Given the complexity of the theory that emerged, diagramming [46] was utilised to emphasise connections. Three of the interview transcripts were co-coded by a second investigator (AW). There was consensus on all codes and so no further co-coding was conducted. The emergent theory was discussed in fortnightly supervision sessions and all co-authors agreed with the representation of the data.

### Member checking

Member checking is an important aspect of qualitative research [47, 48] and critical to the validity and trustworthiness of this study. In this study, multiple member checking processes were undertaken to achieve transactional and transformative validity [49, 50]. These included providing the transcript for review by participants, meeting with a small focus group of participants to discuss initial analysis, and providing all participants with a summary of findings for feedback. The summary of findings was provided in written form as well as a video recording by the researcher that had conducted the research interviews. The aim of presenting the findings in video form was to increase accessibility and engagement as well as appeal to different learning styles [51]. Participants could provide feedback and reflections in open email format or in a more structured short Redcap survey. Providing a structure for feedback was recommended by one of the research participants and cited as a helpful strategy by McKim [47]. Feedback was received from six participants and incorporated into the results.

## Results

Analysis of the data generated two major themes and subsequent theoretical models

1. The theme of ***Misunderstood***, encapsulates the vast majority of eating disorder treatment experiences of autistic young people and parents and included multiple domains leading to the sense of misunderstanding as well as several adverse impacts.
2. The theme of ***Safe and Supportive Treatment*** which includes various sub-themes that depict the characteristics, processes, and outcomes of ideal eating disorders treatment for autistic young people and their families. This theme is based on the reverse of the treatment experiences outlined in theme ‘Misunderstood,’ some treatment elements that were the exception to being misunderstood, as well as the direct suggestions made by participants about how eating disorder treatment services could be improved for autistic young people.

Table 2, seen below, summarises the major themes and themes. Please see Supplementary materials for a table of themes with a sample of participant quotes.

**Table 2:** Themes and sub-themes from the qualitative analysis

Major Theme	Theme
Misunderstood	Problems related to diagnosis
	Siloed expertise
	Limited knowledge and treatment options for non-AN eating disorders
	Misattribution
	Autism not accommodated
	Neuro-normative definitions of eating and recovery
	One size fits all approach
	Lack of consideration of family neurodivergence
	The impact of being misunderstood • Increased burden of case-management and advocacy • Mistrust of health professionals • Identity disruption • Distress and trauma • Setbacks and reduced opportunity for recovery
Safe and supportive eating disorders treatment for autistic young people and their families	Key foundations for treatment • Accessibility • Collaborative multi-disciplinary team • Listening and responding • Compassion and kindness • Tailored approach • Holistic care • Professional humility and curiosity
	Autism-specific foundations for treatment • Neuro-affirming care • Accommodations • Relevant information and resources • Case management and navigation • Working understanding of autism and eating disorders
	Informed by lived experience
	Therapy considerations

### Misunderstood

**Fig. 1:**
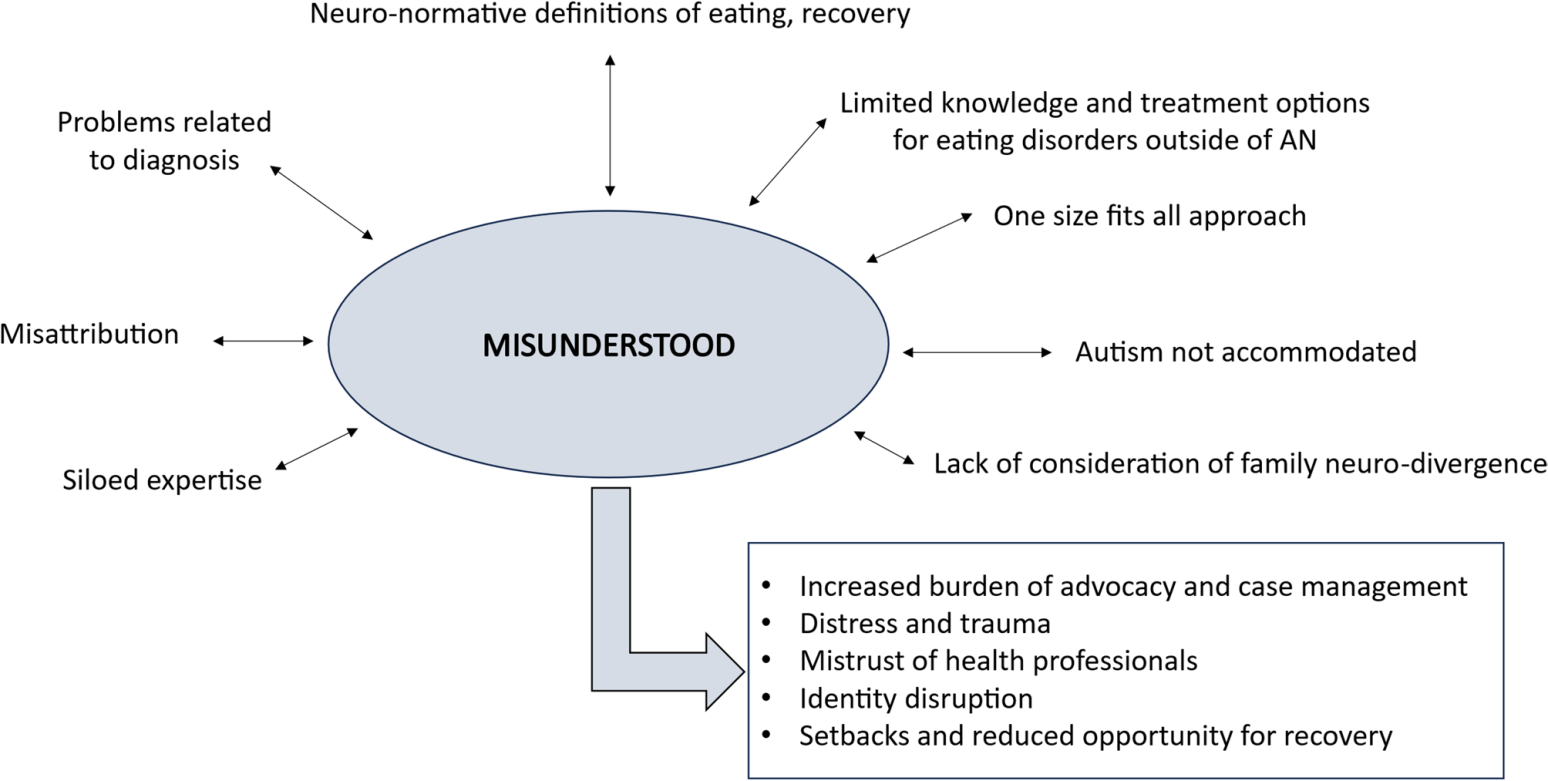
Misunderstood including the eight domains across which a lack of understanding was experienced, and the five adverse outcomes of being misunderstood across these multiple domains

The overwhelming experience of participants was that they and/or their child felt misunderstood. While one participant spoke of generally feeling understood throughout their eating disorders treatment by health care professionals and their parents, all other participants outlined the experience of being misunderstood across multiple domains (depicted above in Fig. 1), and across at least one of the treatment contexts they had engaged with including primary health care, emergency departments, general inpatient care, specialist inpatient care, day programs, and outpatient care in the public and private sectors.

Misunderstanding was found to be a recursive process. When health care professionals do not understand autism, the link between autism and eating disorders, or the young person and what that specific young person needs, this leads to the enactment of one or more of the domains of misunderstanding, for example a one-size-fits-all approach. In turn, these experiences lead to the young person and their parent/carers and other family members being and feeling misunderstood.

“There’s a real lack of understanding even with clinicians.” Gina, Parent.

“I feel like I’m constantly misunderstood.” Emerson, Autistic person (AP)

#### Problems related to diagnosis

A number of problems related to autism diagnosis were identified. These problems are largely related to missed diagnosis or misdiagnosis. A large proportion of participants did not receive their autism diagnosis until accessing eating disorders treatment, and some of these participants did not receive an autism diagnosis until they were adults and had already experienced years of eating disorders care.

Barriers to timely diagnosis were varied, and for many participants, multifactorial. Some participants reported that the cost of autism assessments was prohibitive as autism assessments costs thousands of dollars on top of eating disorders treatment related expenses. Some parents outlined that they had also reduced their participation in the paid workforce in order to care for their child and so financial barriers were even more pronounced.

Access to assessments and treatment services was also identified as a barrier to timely autism diagnosis. Participants cited long waiting lists and geographical distance as barriers to accessing the services that would be able to explore an autism diagnosis.

The barrier to diagnosis that was most commonly cited by participants was what we have described as autism stereotyping. Participants recounted the ways in which some health care professionals, education staff, and family members held a particularly narrow and misinformed view of what autism is and how it is experienced. Participants spoke of situations in which the possibility of autism was raised only to have this dismissed because of this limited view of autism. For instance, Bree reported *“even that psychologist there was like, “Oh no, Bree’s got too much social insight to be on the spectrum.”*

Autistic participants also spoke about their own misconceptions of autism and how this impacted their ability to recognise their own autistic traits, accept their formal autism diagnosis, and explore their autistic identity.

“I’d seen like autism before. I’ve been in like a a like somewhere where there was someone with autism. And they like had like a severe level because of how they interacted with others. So, like when I was diagnosed, I felt like the psychiatrist was wrong because I wasn’t like that.” Chloe, AP.

“Autistic people were in a category of usually nonverbal or stimming or obsessive trains.” Ari, AP.

Parent participants spoke about the challenge associated with supporting their child’s autistic identity when their child had a particular view of autism which did not align with their child’s own autistic traits. For example, Shae stated *“If she could understand what her autism is for her. I feel like that would help a lot because then if I say, “Oh that’s the autism” she has an understanding of what that is and not the extreme end of autism.”* Shae, along with other participants, identified that there is a lack of nuanced resources about autism that match their child’s experience of autism, which also contributes to the perpetuation of autism stereotypes and can alienate autistic young people.

This autism stereotyping and missed opportunities for diagnosis led participants to question their own instincts and meant that they missed out on support and appropriate accommodations, creating an increased eating disorder vulnerability, as well as maintenance of eating disorder symptoms and poorer mental health overall.

“I’m quite surprised that as a kid no one picked up on autism…I think I could have gotten support for that a lot earlier and I think I potentially would have had like accommodations and needs recognised and then enacted and I think that would have heavily supported me in terms of recovery, or potentially even halted the eating disorder itself in its tracks.” Frey, AP.

“I’ve suffered my whole life. And I just didn’t know why…but now [following autism diagnosis as an adult] it saved my life. And explains, cause it explains everything a bit more.” Ari, AP.

“I think if I’d known that I had autism then my ability to kind of identify in myself what were meltdowns and like how I could, what was a more efficient means of coping with them, like I think that would have been improved. I think because I didn’t know what was, how I was feeling and why I was feeling it, like I was kind of more inclined to feed into actually using my eating disorder.” Lauren, AP.

Participants described a very apparent lack of support and resources when an autism diagnosis was finally made. Gina (parent) reported that it it was *“anti-climactic. You get this diagnosis and then it’s like what now? Like there’s a lot they don’t tell you. And I think there’s there needs to be a lot more of a holistic support around parents.”* Chloe outlined that she felt like she was in an experiment when she had an autism assessment as part of her eating disorders care. She stated that she completed the autism diagnostic interview tasks and felt like no explanation was given about the diagnostic test, or about the results when these were provided by the psychologist. The lack of post-diagnostic support is a missed opportunity to help autistic young people and their families feel understood, and have the resources to further understand themselves as well. Participants spoke of the need for eating disorder services to be holistic, and supporting diagnosis was identified as a critical part of this, as outlined by Rachel, a parent:

It would have been really nice if they had come in and said, ‘Oh we’ll do an assessment, you know. Let’s look at all the neurodiversity that there might be going on. Let’s look at what’s actually happening. And you know, let’s look at the whole person.

Another issue of misunderstanding related to diagnosis is that of misdiagnosis. Some of the adult participants spoke of being diagnosed with mental health concerns before their autism was recognised, with these diagnoses subsequently reversed once an autism diagnosis had been made.

“The autism diagnosis has actually been quite recent…she’d taken off my borderline personality disorder diagnosis…all along it was, you know, ADHD and autism.” Lauren, AP.

“There are lots of autistic children and adults like myself who are being misdiagnosed and treated for things are not actually the core primary condition and therefore developing even unhealthier relationships with food when it could have been really supported at the beginning.” Gina, Parent.

As outlined by Gina, misdiagnosis prevents the actual issues being addressed, and thus eating disorder symptoms can become more entrenched and strengthen. With many treatment services targeting particular presenting issues, identifying the primary issue and therefore the most appropriate treatment services is vital.

Unfortunately, even when participants’ autistic traits were recognised, their experience of eating disorder treatment services continued to be dominated by a lack of understanding.

#### Siloed expertise

Participants outlined that it was impossible to find services that understood both autism and eating disorders and the interplay between the two. Participants spoke of their frustration and desperation, knowing that this dual expertise would be vital in the provision of eating disorders care that is supportive and effective for autistic young people.

“What we really need is somebody who is an expert in eating disorders and an expert in autism. And there just isn’t anyone.” Rachel, Parent.

Participants spoke about accessing services that had specialist eating disorders expertise, or specialist autism expertise, but experiencing a lack of crossover of this expertise, and a lack of communication between specialist services. This siloed expertise was to the detriment of assessment, and appropriate and specialised eating disorders care. This lack of dual expertise also resulted in a lack of credibility and trust in treatment teams and eating disorder treatment decisions.

#### Limited knowledge and treatment options for non-anorexia nervosa eating disorders

The other domain in which participants encountered a lack of expertise from services was that of knowledge of eating disorders outside of anorexia nervosa. Participants that had lived or carer experience of eating disorders that were not anorexia nervosa such as avoidant restrictive food intake disorder (ARFID) and binge eating disorder (BED) found that there was even less understanding, resources, and treatment options for these eating disorders.

“It would have been so nice when we sat in front of the GP and said ‘I think we’re talking about ARFID. What do we do?’ That she had a comprehensive answer for me rather than ‘What is ARFID?” Wendy, Parent.

“There wasn’t much in the way of advice we could find at that point, particularly for binge eating in a child. It was there around restricted eating.” Lisa, Parent.

Parent participants seemed particularly shocked at the lack of understanding and treatment options for binge eating disorder and ARFID. They outlined that even specialist eating disorders services struggled to know how to support their children. Autistic young people do experience eating disorders other than anorexia nervosa and these young people and their families experience even further marginalisation and prejudice. One parent explained that their child had been initially denied eating disorders treatment outright because their child had ARFID not anorexia nervosa, and then when the treating team did accept the referral the child received only part of the treatment package that was available to children with anorexia nervosa.

The denial of care and/or limited support and treatment options contributes to an increased burden of advocacy and case management. Parent participants reported having to educate health care professionals about these eating disorders as well as seek their own information and resources and then persistently argue for their child to receive appropriate care.

#### Misattribution

Misattribution was another way in which participants were misunderstood in their eating disorder treatment. Almost all participants spoke about their or their child’s autistic traits being attributed to eating disorder symptoms and behaviours at some stage. Practices such as eating with specific cutlery or crockery, taking small bites, and avoiding some foods due to sensory differences were labelled as eating disorder behaviours despite these practices being life-long. This pathologisation of autistic traits was most pronounced in the inpatient setting. Samantha provided this example of their experience in a paediatric inpatient setting:

“I was also restrained inpatient because I didn’t want to eat the skin of my chicken because I don’t like the feeling, and I’ve never liked the feeling. I ate the rest of my meal and I was trying to explain that it was a sensory issue.” Samantha, AP.

This pathologisation of autistic traits often led to a lack of accommodations and wrongful implementation of consequences and restrictive practices.

A particularly concerning outcome of misattribution outlined by some participants was the way that it changed how they viewed their own autistic traits, and their own internal pathologisation of eating disorder behaviours.

“I was almost conditioned to like pathologise things like that were relative to autism you know, like pathologise them as my eating disorder because everyone else was.” Lauren, AP who experienced inpatient and outpatient treatment.

The pathologisation of autistic traits was not the only way that misattribution was experienced. Some participants outlined that eating disorder symptoms and other mental health issues were conversely misattributed to autism, and thus were not addressed or adequately addressed by eating disorder treatment services. One parent outlined how dangerous misattribution could be in that it led to the denial of serious mental health issues and then a grievous tolerance of these mental health issues.

“To put it bluntly we’ve seen the autism diagnosis literally stop our daughter’s ED team from seeking to identify mental health problems that need treatment and therapy…Being autistic is not being doomed to a life of poor mental health. The messaging should never be that as an autistic person you should just get used to depression, anxiety, or an eating disorder. That is not acceptable. That is not what an autism diagnosis should be used for.” Michelle, Parent.

Misattribution has consequences for identity, for treatment planning and prioritisation, and can lead to distress or trauma if autistic people are reprimanded or given consequences for what is an autistic trait.

#### Autism not accommodated

An overwhelmingly strong theme and domain of being misunderstood, was that of autism not being accommodated across a variety of eating disorder treatment settings including inpatient care, outpatient care in the public and private sectors, and day programs in the public and private sectors. Participants recounted examples of autism not being accommodated in terms of communication practices, brain differences, the treatment environment, and therapy content and processes. Selena identified that even though she provided her daughter’s inpatient care team with written reports, health records, and verbal information about what her daughter needed, these accommodations were not made for most of her inpatient admission:

“There was a lot of information we provided about Maree and her requirements. I suppose we didn’t feel as though there were many accommodations made or a good understanding. That was often a point of tension with the [inpatient] treatment team.” Selena, Parent.

This quote also highlights the negative impact that autism not being accommodated has on the therapeutic relationship between the treating team and the young person and young person’s family. As well as the adverse impact that the lack of autism accommodations in eating disorders care has on therapeutic alliance, it was also described as jeopardising recovery.

“There is a serious reluctance to put in place reasonable adjustments. And I think it’s, I think it’s probably a big contributor to to so many people not overcoming eating disorders.” Michelle, parent, public inpatient and outpatient treatment experiences.

Some participants did experience some useful accommodations that but these were generally limited to one of their treatment settings and were not consistent across all parts of their eating disorders care. For example, Shae shared that her daughter just received the same care as everyone else as an inpatient– that there were no accommodations made, *“in the local hospital…it’s the same rules for everyone.”* But in contrast, when her daughter attended a specialist eating disorders day program, multiple accommodations were made including allowing her daughter to have a quiet space to go when she was distressed, having a separate area to eat rather than eating in the dining room with other patients, and using a quiet tone.

“They were amazing. They’re just so understanding supportive and understand the needs of children with…neurodiversity…they were very softly spoken to her. They were really patient.” Shae, parent.

Accommodations go a long way in counteracting being misunderstood, and therefore there is a need for accommodations to be made more consistently across different eating disorders treatment contexts.

#### Neuro-normative definitions of eating and recovery

One of the specific ways that autism was not accommodated was through the perpetuation of neuro-normative ideals around eating. Neuro-normative eating ideals encountered by participants included sitting down at a table to eat, intuitive eating based on hunger and satiety cues, eating a wide variety of foods, eating with full sized cutlery, and taking large bites of food. This was experienced both in inpatient and outpatient eating disorders treatment settings.

“Yeah, because I said to the dietitian, I don’t think intuitive eating really is not going to be a thing that effective for me. Yeah, it’s actually more anxiety provoking to try and read cues.” Bree, AP, in discussing outpatient treatment.

“They wouldn’t let me have my autistic rules such as I wanted to use the little spoon but they wouldn’t let me because that’s part of the eating disorder…I wanted to cut my toast into eight pieces not two or four as that was part of my autistic eating routine, not my disorder…I wanted to eat the same thing every day because I felt safe with it…but they said ‘No, you have to have a variety of food at different times and different textures and flavours. But because I was autistic, I couldn’t handle that.” Ari, AP.

Participants similarly encountered neuro-normative ideals related to recovery, specifically the markers and timelines of eating disorder recovery. These neuro-normative recovery ideals included recovering within a prescribed short time frame, demonstrating recovery through eating a wide variety of foods and enjoying eating. Alex outlined, that even as she recovered from anorexia nervosa she did not enjoy eating and in fact, never had.

I think for a lot of autistic people food is never going to be the same way, like, you know you see lots of girls who are doing these recovery journeys and they’re like learning food freedom and loving food again and it becomes very deceptive what might be achievable as an autistic person with sensory issues over food…food can be particularly challenging on that aspect you know. As an autistic person I’ve only got so many ‘spoons’ and food seems like a waste of ‘spoons.'

Some autistic participants and parent participants highlighted the pace of prescribed or expected recovery as neuro-normative and therefore not neuro-affirming:

“I just find that the service is not neuro-affirming at all. They didn’t even want to consider it…It’s all rushed. It’s all got to happen now you know, the quicker the better. That’s not the case for everybody.” Rachel, parent.

When eating disorders treatment is based on neuro-normative ideals of food, eating, and eating disorders recovery, it not only perpetuates being misunderstood, it also creates expectations around eating disorders care and recovery that are potentially unattainable, and not attuned to how an autistic person might experience their eating disorder.

#### One size fits all approach

Most participants reported that they encountered a degree of a ‘one size fits all approach’ in at least some elements of their eating disorders care. This ‘one size fits all approach’ was described in different ways by different participants. Rachel (parent) stated that she was told that there was only one form of outpatient treatment available (FBT) and felt like she was treated as “naughty” when she questioned this approach and ultimately did not agree to it. Shae (parent) described the one-size fits all approach as inpatient treatment processes implemented in the exact same manner as neurotypical patients, indicating a lack of tailored care. Many participants outlined experiences of eating disorder care professionals implementing treatment plans without collaboration, curiosity, or nuance.

“Eating disorders are very complex so I’ve never understood why the treatment has to be one monolith when it’s such a complex issue.” Samantha, AP.

“I’ve gone through all the different types of treatment and CBT and introducing foods and all of that but nothing worked because…I don’t think they were treating what they were meant to be treating. It’s not their fault but yeah, they were just treating an eating disorder without the autism factor.” Ari, AP.

Some specific treatment modalities such as FBT and CBT were mentioned as particularly mis-attuned to what was needed as an autistic person by way of attempting to override autistic traits and subsequently increasing distress and placing autistic people in a position of increased masking.

“CBT assumes someone thinks or can think a certain way. What if you’re wired to naturally think in a way that’s different to the way CBT expects you to be able to?” Michelle, Parent.

“I was sent for the outpatient program…it was the Maudsley family-based therapy which I honestly feel like that was more traumatic and did more harm than good…My family all said some really messed up things about me…I mean hearing the rest of my family say those things about me was still probably really bad, like it was really upsetting.” Samantha, AP.

“I think it’s [FBT] not a an autism friendly mode to be honest. Persistent drive for autonomy…we may have some real autonomy drive and that’s the thing about family-based treatments is that they’re taking away your autonomy so much.” Bree, AP.

The adverse effects of mis-attuned treatment included feeling frustrated and misunderstood, the worsening of eating disorder symptoms and overall mental health decline. Some participants also outlined that these treatment modalities were in fact harmful and resulted in treatment-related trauma.

#### Lack of consideration of family neurodivergence

The final domain across which participants felt misunderstood was that of a lack of consideration of family neurodivergence. Parents who identified as autistic (formal or self-diagnosis) spoke of added layers of being misunderstood. All autistic parent participants stated that the eating disorders treating teams had not asked them about their own neurodivergence, and when parents had volunteered this information, there was no follow up conversation, check in, or adaptations made by the clinicians. Autistic parent participants spoke about their caregiving and treatment responsibilities being such that there was no time to pursue their own autism diagnosis or own autism-related and general supports, and they acknowledged the impact that this self-sacrifice had physically, mentally, and relationally. Autistic parent participants stated that they would have benefitted from eating disorders clinicians checking in with them and proactively looking at ways to address the caregiving burden that was exaggerated by having to “fight” (Jackie, parent) the system to put in place autism-appropriate eating disorders care.

Another way that family neurodivergence was found not to be considered was a lack of adaptation of team communication processes. Some participants outlined that the treating team did not make any changes to the way that they communicated with neurodivergent family members. One mother spoke about needing to understand the rationale behind treatment decisions and said that understanding the ‘why’ helped her greatly as an autistic person with ADHD, yet the team involved in caring for her daughter assumed that she was being argumentative when she sought to understand the rationale of treatment plans.

Some parent participants outlined that some aspects of their child’s eating disorder and eating disorder treatment were triggering because of their own current or previous relationship with food.

“Food’s always been a trigger for me, and its it was exceptionally triggering watching the kids go through it too.” Wendy, parent.

Alongside, the potential for triggers was the potential for increased masking due to managing the demands of caring for their child. Rachel outlined that in considering and accommodating her daughter’s neurodiversity she felt like she had to put her own neurodiversity on the “back burner” which in turn led her to feeling overwhelmed and immobilised.

“This is my current struggle– I’ve got to take all the neurodiversity into account but she doesn’t have to take mine. So yeah, it’s really hard. I’ve just got to kind of put that on the back burner…I sit there and I just can’t do anything sometimes because I’m so overwhelmed with everything.” Rachel, parent.

These results suggest that when family neurodivergence is not taken into consideration by eating disorders services it affects all parts of the family system as well as the treatment system.

### The impact of being misunderstood

Being misunderstood across multiple domains was found to be detrimental in a number of ways including:

#### An increased burden of case-management and advocacy

Both autistic people with lived experience of an eating disorder and parents spoke of an increased burden of case management and advocacy due to being misunderstood. Participants spoke of a lack of coordination of care, and having to manage this in conjunction with the demands of eating disorders treatment itself. Some participants received assistance in accessing supports through NDIS (National Disability Insurance Scheme which is an Australian government body that provides funding to people with disabilities to support their goals and improve their quality of life), while others were left to try to apply for NDIS funding and other supports on their own. This was problematic because family resources and time were already stretched in the context of the eating disorder and eating disorder treatment. Alongside coordinating care and sourcing supports, participants also needed to be highly active in advocating for accommodations and tailored treatment. Parent participants used words such as “pushing” and “fighting” to describe the lengths they went to agitate the treatment team and treatment system to get appropriate care for their child.

“I felt like I needed to be there every day pushing, talking, explaining.” Selena, parent.

The burden of this increased case management and advocacy was experienced in the form of frustration and exhaustion or autistic burnout. Both parent participants and autistic participants described the impact that this increased case management and advocacy had on their identity and their working relationship with their eating disorders treatment teams, feeling like they were “annoying” or “problematic” in advocating for their needs or the needs of their child.

#### Identity disruption

The impact on identity was another consequence of being misunderstood. Autistic participants spoke about the impact that having an eating disorder had on their identity, particularly if their illness duration spanned years.

“I wasn’t really connecting with my friends in life. I didn’t really know my own personality. Yeah. I felt like I’ve lost my identity throughout the years.” Chloe.

“It’s just like an identity crisis for years.” Alex.

Participants described that being autistic added another layer of identity difficulties, mostly related to trying to understand themselves in the absence of an autism diagnosis, and living within a neurotypical world and the associated autistic stereotyping and ableism. For example, Bree was told that she *“was difficult, you know, for having those traits.”* This type of ableism and autism stereotyping was cited as leading to denial of authentic self and autistic masking. A number of participants highlighted just how significant the masking was, with many participants speaking of the way that the eating disorder developed in the context of masking. Participants outlined that in trying to fit in with peers, particularly when peers were dieting or focused on their appearance and the thin-ideal, they too began to diet and lose weight to be more like their peers and conform to these damaging social norms.

“I’ve never felt normal or accepted or anything, and I’ve masked my whole life but maybe that part of it [losing weight] made me a little bit normal.” Ari.

“the high masking heavily correlated and perpetuated the disordered eating.” Frey.

One participant stated that she found herself in a “masked” version of an eating disorder. This was perpetuated when the eating disorders treatment providers asked very direct questions about eating disorders symptoms.

“it’s almost like a masked version of my eating disorder…People would suggest me engaging in a behaviour and I went ‘Oh, am I supposed to? Am I supposed to be doing that in order to fit anorexia? So then I would engage in it to be like, am I doing a good enough job of fitting my disorder?” Alex.

Identity was disrupted by the eating disorder itself, and by the difficulties associated with living in a neurotypical world such as stigma and social pressure to engage in autistic masking. Identity was found to be further disrupted by being misunderstood by eating disorder treatment services. This treatment-related identity disruption was experienced in two main forms. Firstly, being misunderstood led participants to question their own sense of self and their own ways of thinking and being. Lauren described that in hospital she was told so many times that her autistic traits were in fact eating disorder traits that she then started thinking that too. She described it as gaslighting herself:

“When I left hospital, I very much started to perceive things like that as…that’s actually is an eating sort of thing, but like I knew it wasn’t because I’d always done it. But you kind of gaslight yourself into thinking that.” Lauren, AP.

Other participants spoke about feeling like common eating disorders treatment practices such as externalisation of the illness meant that they felt “shut down” and were denied their voice or sense of self.

“I didn’t see my eating disorder as separate part to me, as in a different person. And so, it felt like I was being denied my own voice because they kept labelling it as a separate voice and I never understood that. I know it’s helpful for some people to give it a separate but for me it felt like me being shut up.” Alex, AP.

“You’re told things like ‘Oh, you can’t believe anything that they say, you know, that’s not them. It’s just the eating disorder.’ That’s not, you know, that’s not always true. Like sometimes it is them.” Rachel, parent.

The second way that being misunderstood was found to disrupt identity was in the way that it led to increased masking. Participants expressed that they felt compelled to mask within both inpatient and outpatient treatment settings. Participants described that they engaged in masking in inpatient settings to fit in with other patients, to “please” health care providers, and to meet treatment expectations and requirements. In outpatient settings, participants engaged in autistic masking to manage the processes of therapy that was not adapted or accommodated. As one participant highlighted, clinicians failed to recognise the extent and associated fatigue of masking to manage the social processes of therapy sessions– the back and forth conversations, eye contact expectations, and being in room in which the environment had not been adapted. Participants also outlined that some specific therapy models and techniques such as thought challenging, felt like masking was prescribed:

“When it’s CBT I feel like you’re kind of told like keep up that mask and be more social…and push back on thoughts or physical feelings that are telling you like that things aren’t, you know, quite right for you.” Emerson, AP.

Some participants did experience the capacity to “unmask” in certain eating disorders treatment contexts. The characteristics of these eating disorders treatment contexts included specialisation in neuro-affirming eating disorders care, lived experience experts as part of the treatment team, space and support to explore autistic identity, and more choices within treatment.

“I am slowly unmasking.” Ari, AP.

“Like allowing the person to have options…giving me permission that I’m allowed to have a choice…A lot of my masking is about people pleasing. And I find it very hard to put my own needs.” Alex. AP.

#### Mistrust of health professionals

Being misunderstood disrupted identity, and it also impacted trust within therapeutic relationships. In the context of feeling so misunderstood across multiple domains particularly siloed expertise and a lack of accommodations, participants spoke of difficulties trusting the clinicians and teams providing treatment. Participants described that clinicians often seemed to lack knowledge or experience in the interplay between autism and eating disorders, and along with the lack of responsiveness to this interplay, that it was difficult to trust that eating disorder services knew how to support them. This was even more marked for parents supporting a child with an eating disorder that was not anorexia nervosa. These participants outlined that they desperately needed professional expertise and support but even finding a health care professional that understood binge eating disorder or ARFID was extremely difficult. This was a further gap in knowledge and credibility and therefore trust in the treatment process was unfounded.

One parent participant who is autistic herself described that she was unable to trust her daughter’s treatment team because they did not provide a rationale for her treatment plan and therefore did not provide what she needed in the process. Understanding the thinking and intentions that were informing her daughter’s treatment plan was important as a parent, and even more important as an autistic person in that understanding facilitates certainty.

“Don’t just tell me something. I need to understand why. And it’s not challenging you, it it’s I need to understand why because if I understand why you’re doing certain things and what the goals are I can actually use my understanding to try and fill the faps or to provide extra information, and not just that, it reduces uncertainty for me like that. Uncertainty is so dysregulating, and it’s alarming, and it it means I can’t trust you because I don’t know why you’re doing what you’re doing.” Michelle, parent.

Participants also spoke of the mistrust that occurred when treatment was applied in a one-size-fits-all approach. Participants described feeling like they were treated like “just a number” when particular treatments or inpatient processes were recommended or commenced often without meaningful time to get to know the young person. In the absence of a holistic, tailored approach, participants found it difficult to trust the recommendations made by staff, particularly if recovery rates of the recommended treatments or programs were generally low or low for autistic people specifically. Conversely, for participants that did have an experience of being listened to and having some aspects of care tailored to their needs, there was the opportunity for rapport and a strong ongoing therapeutic relationship to form.

“We were very lucky initially with our GP. She was just beautiful, beautiful connection with Liv.” Jackie, Parent.

Mistrust of health professionals was experienced at both a knowledge level, and at a relational level. Credibility of treatment teams and treatment plans was low when there were significant gaps in clinician knowledge and experience, and mistrust in the therapeutic relationship resulted from a lack of nuanced, personalised care.

#### Distress and trauma

One of the most pervasive effects of being misunderstood across multiple domains was the resulting distress and trauma. This distress and trauma was experienced across a spectrum by participants. Some participants described feeling lost *“My husband described it like falling down a hole, like Alice. Grabbing things and trying to understand”* (Selena, parent). Others spoke to the experience of feeling overwhelmed or burnt out. In describing her inpatient care, Clara stated.

“The structure is nice somethings and can also be a bit overwhelming because it’s so regimented. Very contradicting to what I was saying but there’s an in-between.” Clara, AP.

For some participants, the experience of being misunderstood by eating disorders treatment experiences was incredibly traumatic. Jackie described that her daughter is so traumatised by the eating disorder hospital admissions that she does not want to have her own children because that would mean she would have to go to hospital to give birth.

“We’re out of hospital because we’re so traumatised we can’t go back there, you know. So, it’s it’s not success, that’s not success. The fact that my daughter won’t, doesn’t ever want to walk into a hospital or have kids or stuff because she’s so, that it’s meant to be a place of care, you know, and it’s not, which is just not right, is it?! Jackie, parent.

Neurodivergent parents had an added layer of potential distress and trauma in that it was not only their child being misunderstood but also themselves. Alongside this, parents may need to care for their child in a way that is contrary to what they themselves need as an autistic person.

#### Setbacks and reduced opportunity for recovery.

Ultimately the impact of being misunderstood across multiple domains were setbacks in treatment and reduced opportunity for recovery. Without adequate autism-specific supports and accommodations, with compromised therapeutic alliance, with disruption to identity, and with increased distress and trauma, participants outlined that their recovery journey was adversely impacted. For some participants this was experienced as an escalation in eating disorder symptoms, as described by Selena:

“Maree had been changed from the room she was in for the last five weeks. They suddenly came in and said you’ve got to go to this other room. So pack up all your…stuff. And they had done it while she was on her own. They told her with no notice…And she was distraught. And that upset her for days and days and days. It could’ve been done so differently. Like they could have waited until I was there, given a bit more notice, even if it was just an hour. I could have helped to pack up all those little things, we could have gone into the new room. And it seems like a silly little thing but it set her back. Like she stopped eating, and it was really not done very well.” Selena, parent.

Other participants experienced setbacks in the form of increased autistic burnout, directly as result of being misunderstood and unsupported. For example, Emerson reported that after a number of life stressors alongside unhelpful support with an eating disorder, they found it difficult to leave the house and engage in normally enjoyable activities. Emerson outlined that it took a long time to recover from what they now know was autistic burnout.

Autistic participants that were not recognised as autistic until adulthood attributed the development and ongoing maintenance of eating disorder symptoms to this delay in getting an autism diagnosis. The lack of recognition of their autistic identity meant that there was increased vulnerability of eating disorder symptoms developing as well as a lack of informed supports which led to maintenance of the eating disorder and a prolonged illness. Some participants noted that when they did eventually receive their autism diagnosis it aided recovery quite dramatically:

“I went through the process and then yeah, I was diagnosed with level 3 autism and ADHD. At the start– just confronting and scary and confusing but now, like a year later, it saved my life. And explains, because it explains everything a bit more. And I went from suicide, serious suicide attempts to and thoughts of it daily, to not ever wanting to do it because I am me for a reason.” Ari, AP.

The impact of being misunderstood across multiple domains impacts autistic young people in different ways and to varying degrees depending on their experiences but ultimately the impact affects the individual and their sense of self and safety, the family system, therapeutic alliance, and the course of treatment.

### Safe and supportive eating disorders treatment for autistic young people and their families

Participants were passionate about their treatment experiences being heard and making suggestions about how eating disorder treatment could be improved for autistic young people and their families. These accounts and ideas have shaped the second major theme and theoretical model, that of ‘safe and supportive eating disorders treatment for autistic young people.’

**Fig. 2:**
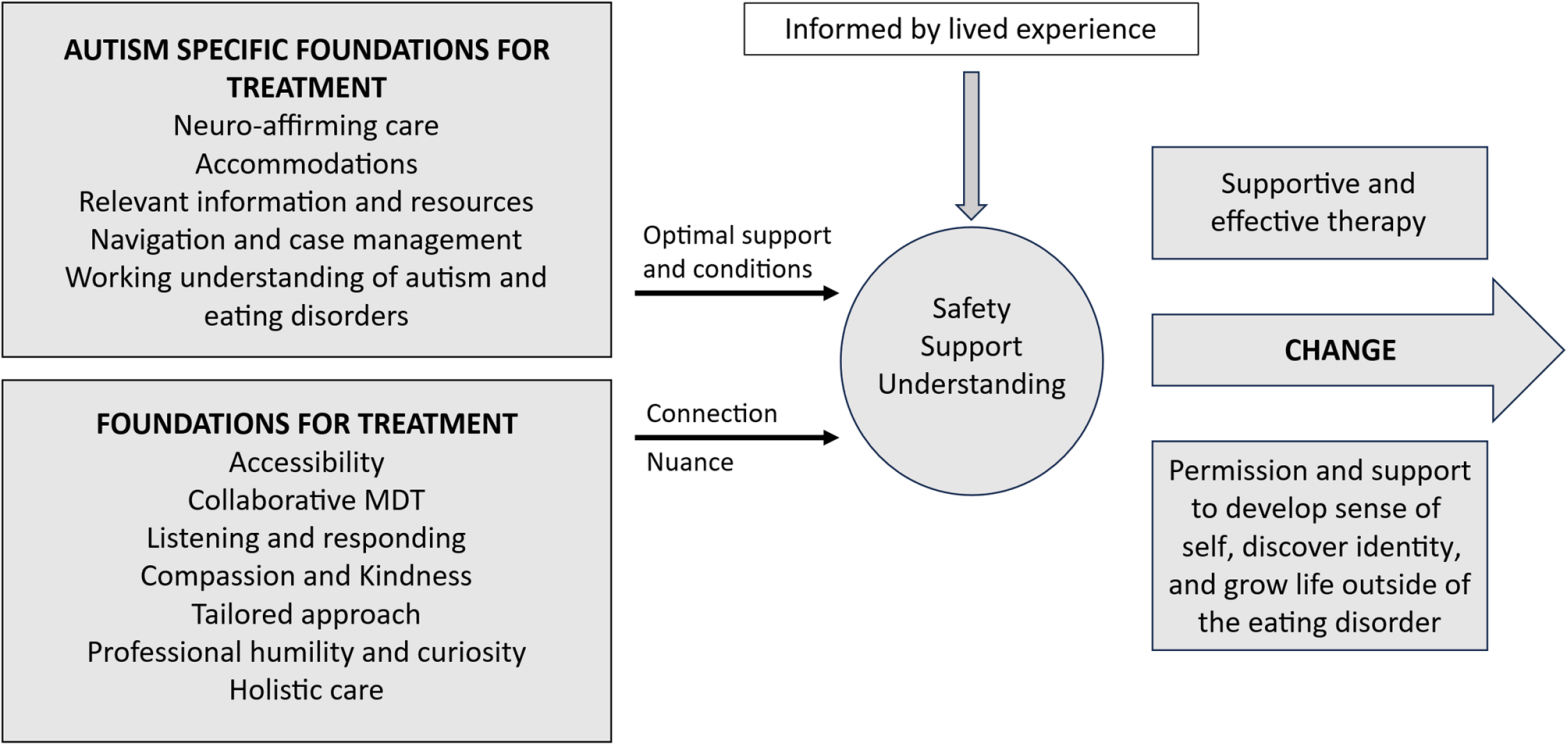
An outline of safe and supportive eating disorders treatment for autistic young people and their families. Safe and supportive treatment is informed by lived experience and built on general foundations for treatment that promote connection and facilitate nuanced care, as well as autism specific foundations for treatment that provide optimal support and conditions

Several foundational elements of treatment were identified as crucial in the provision of safe and supportive eating disorders care for autistic young people. These foundational elements can be grouped into general foundations for treatment, and foundations that are specific to autism as outlined in Fig. 2.

#### General Foundations for Treatment

The key foundations for treatment are those elements that provide a platform for engagement in treatment, the development of rapport, and ongoing therapeutic alliance. At the centre of these key foundational elements of treatment is connection between the young person, their family, and the treating team. While participants were asked directly about autism-related aspects of care, all participants spontaneously spoke of characteristics of care and of clinicians that were not specifically related to autism too. Accessibility was one of these foundations for treatment. Participants highlighted that eating disorders treatment needs to be affordable, timely, and equitable.

“The elephant in the room has also just been finances. The eating disorder appointments are just really expensive.” Lisa, parent.

“And then you’ve got the wait lists that are closed because there’s just too many people waiting. So it is really, it’s actually impossible to get the kind of help that you need.” Wendy, parent.

With many participants living regionally or rurally, geographical barriers were mentioned frequently including a lack of local services and the subsequent time taken to travel back and forth from metropolitan areas.

“We’ve had a lot of struggles because we’re in regional…we don’t have the facilities and resources down here.” Jackie, parent.

“It’s an hour to get there, an hour for the appointment, and an hour to get back.” David, parent.

While accessibility speaks to the logistical aspects of eating disorders care, all of the other key foundations for treatment outlined by participants were more relationally orientated. The characteristics of care and of clinicians that were reported as critical to safe and supportive treatment included being listened to and understood, collaboration between the team and the young person and their family, professional humility and curiosity, and a holistic, and a tailored treatment approach. Participants highly valued being heard and understood and being treated with compassion:

“Listening, yeah hearing her voice. Not treating her like an illness and a patient and a number.” Jackie, parent.

“As time went on, I felt like we were really being listened to.” Selena, parent, speaking about inpatient treatment

I just wish clinicians understood how distressing it is and how recovery can be harder than maintaining an eating disorder. Yeah, I just wish there was more compassion. Bree, AP.

Participants also valued collaboration and autistic participants specifically spoke of the desire to be included in treatment decisions, particularly as this was recognised as an adaptation of standard practice:

“Sometimes the health like the medical and the medical people they say to take all the control. But Mum and Dad, they still give me some control. So, I am having a say in what I’m doing which I think is very helpful and very much good for the autism side of things.” Clara, AP.

“Including the kid in treatment. And you know, letting them take an active role because I know a big thing in eating disorders is control.” Samantha, AP.

Participants perceived humility and curiosity to be key clinician characteristics that fostered this collaboration and compassion. Humility was demonstrated when clinicians spent time being genuinely curious and interested in getting to know the person and family. Humility was seen to facilitate a deeper understanding of the young person and what they need from eating disorders care. Participants outlined that humility is also evident when clinicians acknowledge and apologise for mistakes or gaps in knowledge, paradoxically increasing the clinicians’ credibility with the young person and their family:

“I would rather a GP now that came in and said ‘I don’t know all about eating disorders but I’m willing to go on the journey with you and learn with you.’ Like I love when people say ‘I don’t know everything because they don’t and they just want to listen to you. And you know they’re the best people.” Jackie, parent.

Professional humility and curiosity provides a strong foundation for nuanced and holistic care. Participants strongly advocated for standard eating disorders care to be adapted and tailored to the needs of autistic young people. Not only are the needs of autistic young people different to neurotypical young people, but each autistic young person is different and therefore has different needs as summarised by Clara (AP) “*although autistic people have very similar traits each one’s different.”* Participants repeatedly spoke about how vital it is to personalise and tailor eating disorders care for each individual. These individual differences were seen even within the research interviews, for example one participant found externalisation of the eating disorder “life changing” (Bree, AP), and several other participants reported that externalisation felt like being told to deny their/their child’s voice (Alex, Samantha, Jackie, Rachel).

Participants also advocated for holistic eating disorders care. Participants described holistic care as understanding the broader identity and context of the young person, and focusing on non-food related issues.

“I think we’re trying to treat her as if the only concern was the eating disorder. They didn’t treat it as with a holistic approach taking into consideration the other concerns.” Lisa, parent.

Participants outlined that it was helpful when eating disorder clinicians supported them or their child with issues such as connecting with friends, identity, feelings of needing to be in control, social anxiety, depression, trauma, and school related issues.

“We started with the third psychologist who understood that we needed to do things a little bit differently and try to work with Mia on building her connections back…hardly ever spoke to her about food. It was more about building the connection back with some friends and gradually, as that started to come back, she started to, you know, eat more and like she had something to work for.” Rachel, parent.

The clinician characteristics of compassion, humility, curiosity, and genuine listening, as well as treatment being accessible, tailored, and holistic provide a strong relational connection between autistic young people, their families, and treating team. In turn, this connectedness promotes a good understanding of the young person and their individual needs which provides a solid platform for nuanced care.

#### Autism-specific foundations for treatment

Alongside general foundations for treatment, participants identified a number of autism-specific foundations that are critical for eating disorders treatment to be safe and supportive for autistic young people. The autism-specific foundations highlighted by participants included a working understanding of the interplay between autism and eating disorders, accommodations, relevant resources, and proactive case management and advocacy. When these autism-specific foundations of eating disorders care were in place, treatment was seen to be neuro-affirming. As outlined by Emerson, when eating disorders care is neuro-affirming it is also safe:

“The eating disorder special programs– there’s only like one or two and they’re very hard to get into and then you don’t know if they’ll be accommodating to neurodivergent people, particularly autism. So, you’re kind of always going into spaces not knowing if they’ll work for you and having to be on high alert and so to have a program that you know would be like actually safe would be incredible.” Emerson, AP.

First and foremost, participants stated that eating disorders services and clinicians need to have a working understanding of the interplay between autism and eating disorder. Participants whose child had an earlier autism diagnosis and were linked in with autism-related services had difficulties finding health professionals that understood eating disorders, and participants that were predominantly involved with eating disorder services had difficulties finding health professionals that understood autism. Without a working understanding of both autism and eating disorders, and the interplay between autism and eating disorders, the other autism specific foundations of care cannot exist.

“to actually understanding the not just eating disorders and not just autism but both of them and how they come together because I think there’s not enough understanding.” Samantha, AP.

Participants relayed that relevant resources on autism and eating disorders were also lacking. Many participants spoke about difficulty accessing information and resources about autism, about autism and eating disorders, and about eating disorders such as ARFID and BED. Resources were difficult to access because their either did not exist, or resources that did exist did not seem relevant to participant experiences of autism.

“When I knew it was autism and that made a huge amount of sense to me personally and for her so. But usually this is this whole catalogue of emotions going on and misinformation is the worst part of that. And so having a trusted one point of contact with that information that’s correct.” Wendy, parent.

“Most of the online resources that I see don’t fit Chloe’s case.” Shae, parent.

Participants therefore outlined that eating disorders treatment services need to provide relevant and up to date information about autism and eating disorders. A couple of parent participants relayed good experiences that they had with a specialist neuro-affirming eating disorders service in the private sector. The resources provided by this service was seen to be a very significant part of what was helpful about this particular service. The provision of such information and resources empowers autistic young people and parents and alleviates some of the burden of case management and advocacy.

Alongside the provision of relevant information and resources, participants stated that the burden of care and advocacy would be decreased if eating disorder clinicians took a more proactive approach in providing support and case management.

“They’re operating as silos, completely independent of each other so they don’t know where they should start, where they will stop. There’s no communication at that interface. Yeah, a coordinator would help too. Make sure there’s no gaps.” David, parent.

It is the proactive approach that participants highlighted as critical in ensuring that eating disorders care is safe and supportive.

“It would be nice if people were a bit more proactive.” Wendy, parent.

Participants outlined the need for proactive support from health care professionals with case management and navigating systems such as NDIS.

“You’ve got to have NDIS really to cover the costs. And that’s you know another another nightmare minefield trying to. We haven’t even started that because I don’t even know what to ask for…that would be nice too to have somebody helping with NDIS.” Rachel, Parent.

Navigating systems such as NDIS is complex and time consuming and parent participants reflected on what a difference it makes when this burden is shared with or carried by the treating team. The impact of having proactive support was seen to be helpful in practical day-to-day sense, and was also helpful in a relational sense. The experience of being proactively cared for strengthens the therapeutic alliance with the treating team.

“We had [day program team] help us with her NDIS application because that just looks too difficult. They were amazing. They’re just so understanding and supportive.” Shae, parent.

Participants spoke about the way that the provision of accommodations also made them feel seen which led to a helpful sense of connection with the health care team. This sense of connection with the treating team alongside the increased accessibility of care when accommodations are implemented facilitated improved treatment experience and effectiveness. Many of the accommodations were described as little or inexpensive yet make a big difference.

The range of accommodations mentioned by participants was broad and included sensory, communication, environmental, routine, and social accommodations. All of the accommodations that were mentioned by participants are collated in table 3 below.

**Table 3:** An outline of autism accommodations that were identified as helpful by participants

Type of accommodation	Accommodation
Sensory	Providing fidgets
	Clinician using fidgets as a way of modelling and giving ‘permission’ for fidgets to be used
	Use of noise cancelling head phones
	Soft, fluffy bathrobe for gown weighing rather than hospital gown (with robe weighed for comparison)
	Proactive conversation about stimming and support to stim
	Weighted toys and blankets
	Aromatherapy
	Warm baths and showers as sensory soothing
	Wearing headphones
	Hard pressure items
	Looking at ways to reduce noise
	Quiet space to wait in the Emergency Dept
Food and eating related accommodations	Allowing same foods
	Allowing more packaged foods because these are predictable
	Less insistence on variety e.g. Support to have the same breakfast every day
	Permission to use own cutlery or crockery
	Allowing use of small spoons
	Not focusing on fear foods. Starting with same foods.
	Capacity to take small bites
	Bringing in own food from home to increase familiarity and avoid poor quality hospital food
	Recognition of foods that have never been eaten and removing these from hospital meal plan
	Permission to not have to sit at the table to eat
	Allowing choice and agency around all or some food decisions
	Longer dislikes food list
	Understand fluctuating capacity (spoon theory or energy accounting) and adjust meals accordingly
	Designated cupboard or box for food and snacks that no one else is allowed to access
	Using cooking as an entry point into getting more comfortable with food (ARFID)
	Focus on habitual/scheduled eating rather than intuitive eating
Communication	Use of visual aids and print outs
	Communication diary (for use with inpatient team)
	Signs for inpatient room e.g. ‘Please don’t enter, I am resting.”
	Use of silence and pauses in therapy sessions
	An outline or agenda for therapy sessions to increase predictability
	Explaining the ‘why’ about treatment or requests
	Providing relevant information alongside space and time for the young person to interpret the material for their own situation
	Notice of change
	When a change does have to take place, e.g. Changing rooms, ensuring that a support person is present
	Clear, direct communication
	Reduced number of health care professionals in the room in MDT meetings with young people and their parents
	Phrasing questions so that only require a ‘yes’ or ‘no’ response is required, or thumbs up/thumbs down, particularly when distress or overwhelm is high
	Reduce hospital jargon
	Permission not to make eye contact
	Increased focus and conversations on interests
	Liaison with school
	Allow more time for communication and different processing speeds
	Listening to podcasts by experts by experience
	Present the facts and offer choices based on these facts
Environmental	Own inpatient room
	Room near nurses or other patients for ease of connection
	Dim lighting
	Not sitting directly under a light
	Space to be alone and wind down
	If there is need to share a hospital room, sharing with another neurodivergent person
	Access to interests
	If in a shared room, make sure other patients have similar age/reasons for admission e.g. Not in a room with elderly dementia patients
Family neurodivergence	Facilitate/organise assessments for other family members
	Assess risk of eating issues for all neurodivergent family members
	Explain the ‘why’ of treatment
Treatment and therapy related	Longer duration of treatment
	Individual therapy time as well as family support
	Focus on special interests and incorporating these into treatment
	Being weighed by the same person each time
	Consideration of use of externalising language (some participants found externalising language helpful, but others found externalising language unhelpful therefore consideration and collaboration is needed)
	Young person to be part of the sessions with the dietitian
	Access to a broader MDT including OT and Speech Therapists
	Not setting homework - to reduce autistic burnout
	Consistent therapist (not students on placement that will have limited time frame)
	Access to peer support worker
	Doing art or other things alongside conversations in therapy
	Have identified goals for treatment and therapy
	Break goals into smaller goals/steps
	Focus on underlying issues and trauma
	Assessment and consideration of co-occurring conditions such as Ehlos-Danlos syndrome
	Recognising that body image issues may not be relevant
	Less emphasis on time with friends and being independent if those things are difficult from a neurodivergence perspective
	Work on social anxiety
Other	Low demand approach particularly after difficult situations
	As much choice and control as possible

These autism-specific foundations facilitate the optimal conditions for autistic young and their families to be able to access treatment and experience treatment as more effective. The autism specific foundations ensure that care is neuro-affirming and adapted to meet the specific needs of autistic young people with eating disorders. The autistic specific foundations also create an increased sense of being seen and understood by eating disorders treatment teams, thus strengthening connection and therapeutic alliance. In combination, the key foundations for treatment and the autism-specific foundations for treatment create safety and understanding that facilitates nuanced and effective eating disorders care of autistic young people. Safety, support, and understanding provide a platform for change to occur, as depicted in Fig. 2. When these foundations are in place, specific therapy content and processes are experienced as more effective and more likely to support recovery. 

Participants placed high value on the inclusion of lived experience expertise. Knowing that treatment was informed by lived experience perspectives or being able to meet people (clinicians or peers) who were neurodivergent and had lived experience of an eating disorder was seen as incredibly helpful.

“Lived experience is really important, and that your clinical background will only be benefitted by learning from lived experience, and that you don’t need to hear it or disregard it.” Gina, parent.

“I think peer support is good for both the child and the parent.” Frey, AP.

“Just listening to other people talk about what autism entailed for them and I was like, ‘Oh well, I experienced that too, so that kind of made sense.” Samantha, AP.

Many participants expressed a desire to see much more lived experience expertise incorporated into eating disorders care for autistic young people.

In regards to the format of therapy, participants highlighted that a combination of individual and family approaches would be most suitable, with sessions needing to be face-to-face rather than telehealth sessions. Several suggestions were made about what might be useful to focus on in eating disorders therapy including understanding the general and person-specific interaction between autism and eating disorders, the role of control, identity work including autistic identity, preventing and managing autistic burnout, and growing advocacy skills. It is worth noting that furthering advocacy skills cannot be done in isolation from addressing systemic issues.

Being misunderstood is central to the current eating disorder treatment experiences of autistic young people and parents. To move from treatment experiences defined by misunderstanding to treatment experiences defined by safety, support and understanding, key foundations of treatment combined with autism specific foundations of treatment need to be implemented.

## Discussion

The aim of this study was to understand the adolescent eating disorder treatment experiences of autistic people and parents of autistic young people. Utilising constructivist grounded theory methodology, data was collected through semi-structured interviews and analysed concurrently, with theory drawn directly from participant accounts. Two major themes emerged, that of (1) Misunderstood and (2) Safe and Supportive Eating Disorders Treatment for Autistic Young People and their families. These findings provide valuable insight into the treatment experiences of autistic young people and parents of autistic young people, particularly in relation to the domains across which this lack of understanding occurs and the subsequent adverse effects of being misunderstood. Building on these treatment experiences, a range of treatment recommendations were made by participants, developing a theory of what constitutes safe and supportive eating disorders treatment for autistic young people and their families.

The experience of being misunderstood was found to be a common occurrence. This is consistent with much of the qualitative research focused on autistic adults with lived experience of eating disorders [19, 20, 52–54] as well as carers of autistic people with eating disorders [19–21, 55]. It is concerning that this too is the experience of adolescents and their families, particularly when adolescence is such a formative stage of life socially, cognitively, emotionally, and physically.

Building on the general concept of a lack of understanding, this study found that being misunderstood occurred across eight domains including issues related to diagnosis, neuro-normative definitions of eating and recovery, stereotyping, family neurodivergence, siloed expertise, lack of autism accommodations, non-anorexia nervosa eating disorder diagnoses, and the application of a one-size-fits-all treatment approach. A lack of recognition of autism accounts for some of this misunderstanding with many people only receiving an autistic diagnosis as part of their eating disorders treatment, and often after many years of eating disorders care [13, 28, 56]. Yet, even when an autism diagnosis was made, misunderstanding continued to be the most common experience for participants. The range of domains of being misunderstood were far-reaching and occurred in a variety of treatment contexts including public and private care, inpatient and outpatient care, and day programs. The identification and exploration of these domains is important as the specificity of these spheres of misunderstanding will enable targeted treatment enhancements and improvements. In this way the current study, aside from focusing on the experiences of adolescents, extends past research by ‘unpacking’ what leads to the overall experience of being misunderstood in an eating disorders treatment context.

Given the breadth of this lack of understanding a number of harms were outlined by participants. Common adverse impacts of being misunderstood were identified and include an increased burden of advocacy and case management, mistrust in health professionals, identity disruption, distress and trauma, as well as setbacks and reduced opportunity for recovery. These are important experiences to highlight as they provide a strong justification for the consideration of treatment adaptations to prevent these harms in the future.

The impact of being misunderstood on identity is particularly important to consider given that identity formation is a critical aspect of adolescent development [57]. Feeling misunderstood by their treatment team may contribute to young people having difficulties in understanding themselves. Children and adolescents need a secure attachment base to navigate developmental tasks and develop a sense of self and autonomy [58]. While the relationship with the caregiver is the main attachment base, clinicians also have a role to play in providing security through repeated experiences of support and understanding in treatment and care settings [59, 60], especially during these formative years.

Identity is also related to both eating disorder vulnerability and recovery [61–63]. Verschueren et al. [64] outlined a recursive process between identity and eating disorders. They found that problems in forming a sense of identity increased eating disorder vulnerability and symptomatology, and that the presence of eating disorder symptoms decreased identity synthesis– the process of developing a more stable sense of self and core values, beliefs, and priorities [65]. In looking specifically at adolescent identity and eating disorders, Verscheuren [66] found that adolescents who struggled with identity tasks had a greater risk of developing eating disorder symptoms over time, and that patients with eating disorders experienced profound identity issues. One participant from the current study summarised the experience of having an eating disorder as “just like an identity crisis for years.” Given the link between identity issues and eating disorder vulnerability and maintenance it is vital that autistic young people have experiences of being understood and accepted, and being supported to explore their own identity including their autistic identity. Missed opportunities for diagnosis, misdiagnosis, misattribution, and autism stereotyping are barriers to identity synthesis.

Alongside identity crises that are typically associated with eating disorders, autistic young people experience a further challenge to their identity formation and sense of safety due to the stigmatisation, pathologisation, and marginalisation of autistic people. This can lead to the conscious or unconscious suppression of autistic traits and autistic identity known as masking [67]. Masking has been described as a trauma response [67] or a way to avoid ongoing stigmatisation [68, 69]. Masking is associated with autistic burnout, increased risk of suicide and other mental health difficulties [70], and has been found to contribute to identity confusion and have a profoundly negative impact on identity [71]. Masking was identified as a common way participants in this study attempted to manage the ongoing lack of understanding they experienced from their eating disorder treatment providers. Participants outlined the way it impacted their identity and well-being including doubting themselves, not being able to accept their autistic identity, feeling like they were being ‘difficult’ if they requested any accommodations or increased support, and feeling exhausted or burnout.

A compounding factor associated with being misunderstood is the detrimental impact it has on relationships [72], and in the case of eating disorders treatment, therapeutic relationships. For example, when autistic traits were pathologised as eating disorder behaviour, participants expressed that this led to mistrust and a breakdown in the relationship with the treating team. Participants also frequently recounted the ways in which they felt ‘unseen’ when simple autism accommodations such as being allowed to eat with smaller spoons, were not made. These relational breaches made it difficult for participants to connect with their treating team, acting as a barrier to recovery. This aligns with one of the major findings of Rankin et al.’s [73] systematic review of lived experienced research in inpatient care for anorexia nervosa which found that when patients had an experience of being misunderstood by health care professionals it created a milieu of opposition and prevented change. In contrast, they found that when patients had an experience of being seen and heard there was an increase in recovery-orientated behaviour. It is well known that the therapeutic relationship, including therapeutic alliance, is pivotal in treatment experiences and outcomes across multiple treatment settings [74–77] and this has also been specifically evidenced in eating disorder treatment outcomes [78, 79]. In a systematic review of intensive eating disorder treatment Webb and colleagues [80] found that characteristics such as collaboration, kindness and warmth, being heard and seen, treated as a person not an illness, and tailored holistic care were associated with eating disorders recovery. In the current study clinician characteristics of listening and responding, compassion and kindness, tailoring approaches, humility, and emphasising holistic and collaborative care were seen as essential in fostering the therapeutic relationship and aiding the treatment process. Thus, when there is a lack of understanding from the eating disorder clinician, it not only affects the autistic individual themselves, it can also interfere in the effectiveness of treatment itself by reducing the therapeutic alliance.

Given the repeated and broad experiences of being misunderstood, the relational context of eating disorders treatment for autistic young people must be defined not only by therapeutic alliance but first and foremost by relational safety. Podolan et al. [81] provide a definition of relational safety as involving “ a trusting, empathic, non-judgmental, and confidential therapeutic relationship that encourages clients to express themselves freely and promotes their well-being.” While there are differences in ideas of what specifically constitutes relational safety between clinical scholars, there is agreement that no matter the specific construct or modality, therapeutic change can only occur in the context of relational safety [81]. This corresponds with what was reported by participants in this study– safety is paramount to overall well-being and to eating disorder treatment experience and outcome.

Alongside treatment characteristics that promote connection, it is also vital that autism-specific supports and adaptations are implemented to optimise support and further strengthen the therapeutic alliance. This study confirms the need for adaptations such as those applied in the PEACE pathway [27, 28], including proactive implementation of accommodations such as access to sensory tools and adjusted meal plans; the provision of relevant information and resources; and clinicians that have a working understanding of autism and eating disorders, and highlights the relevance of these accommodations for children and adolescents. However, this study has identified additional adaptations to treatment, specific to the paediatric context.

One additional autism-specific adaptation that was identified by participants in this study, particularly parent participants, was the need for eating disorder treatment services to provide more proactive case management and system navigation. Examples cited by participants included assistance in applying for and liaising with insurance schemes (in Australia, NDIS), coordinating outpatient appointments to reduce travel and time spent at treatment centres, advocating in educational settings, arranging autism and ADHD assessments for patient’s family members, coordinating communication between clinicians across services, and checking in with parents about their own well-being. Provision of such proactive case management and navigation has been recognised as a valuable service improvement in autism support service settings [82]. In this current study it was identified that proactive case management and care navigation would be important in alleviating the increased burden of advocacy that is placed on autistic young people and their families, and which they feel is a direct consequence of systemic misunderstanding.

Another additional aspect of eating disorder treatment identified in this study is consideration of family neurodivergence [29]. Parents who identify as neurodivergent were found to be even more affected by being misunderstood as they had to manage and challenge the lack of understanding and lack of tailored care for their child, as well as the ways in which they themselves (and other family members) were misunderstood. Parents in this study who identified as autistic or possibly autistic emphasised that they would have benefitted from such changes as communication style adaptations wherein more extensive treatment rationales were provided, as well as family meal plans as opposed to individual meal plans to accommodate multiple differences in eating. Parents’ own experience as a neurodivergent person strengthened their understanding of their child’s needs but this insider-knowledge also potentially added to the distress when their child’s needs were not being considered or met. The result was increased masking, autistic burnout, and distress or trauma, alongside reduced time to access their own supports and adaptive care practices. Thus, for safe and effective eating disorders treatment it is imperative that care is tailored not only for the young person but also for the family - utilising family expertise and not without ensuring that care teams are providing proactive support, case management and advocacy.

There remains a great deal to understand about how therapy itself needs to be tailored for autistic people experiencing eating disorders. In the comprehensive report, ‘Neurodivergence and Eating Disorders, A Stepped Care Approach’ by Cobbaert and Rose, the challenges of traditional eating disorder therapies for neurodivergent people are highlighted including perpetuation of neuro-normative ideals, pathologisation of autistic traits, and a failure to recognise the specific aetiological considerations of eating disorders experienced by autistic people [30]. Several participants’ experiences of traditional eating disorders therapies such as Cognitive Behaviour Therapy and Family Based Treatment confirmed these issues. There were specific treatment components that were found to be unhelpful by most participants, such as the FBT premise that parents take full responsibility and control of food and eating decisions or the CBT focus on challenging thoughts that may actually be protective or well-founded, but it was the assumptions and outright prescription of these therapy models without collaboration and consideration that was found to be most damaging. Participants who found aspects of traditional eating disorder therapies helpful did so in the context of experiencing clinician humility and a collaborative tailored approach, thus highlighting the importance of therapeutic process and relational safety.

Safety was also found to be established through the centrality of lived experience in eating disorders treatment. This study highlighted the value of lived experience leading and informing research, clinician education, peer support, implementation of autism specific accommodations and the development of treatment adaptations. Research participants emphasised that it would be easier to trust the system if the system or service had been informed by autistic people. Epistemic humility is vital in the prioritisation and meaningful integration of lived experience [30]. Thus, humility is an important characteristic of both clinicians *and* systems to foster inclusive, safe, and supportive eating disorders treatment. Epistemic humility and the inclusion of autistic people in research, service development, service implementation, evaluation, and peer-mentoring roles needs to be an ongoing priority and process.

### Strengths and limitations

A key strength of this study is the centrality of the autistic voice and lived experience. At each stage of the research process the autistic voice was prioritised including the research conceptualisation, research design, accessibility accommodations, and multi-tiered member checking practices.

Paediatric eating disorder treatment experiences were explored from the perspective of autistic people as well as parents, facilitating a depth of understanding and role-specific treatment considerations. Further, the perspectives gained in this study included those from a variety of geographical locations within Australia, both autistic and non-autistic parents, as well as a range of eating disorder diagnoses and treatment contexts. This contributes to the study’s strong information power [83].

The study demonstrated rigour through multiple member-checking processes, data triangulation, the involvement of two authors in coding and data analysis, and reflexivity.

This study also has several limitations. There was an absence of perspectives from people from culturally and linguistically diverse backgrounds, and while data saturation was reached across almost all domains, it would have been beneficial to have more fathers or some autistic boys and men participate in the study to be able to compare treatment experiences across genders. It is likely that there is potential for volunteer bias, with participants being highly invested in seeing change in eating disorder treatment for autistic young people. Finally, given the majority of autistic participants were not currently adolescents, there was a reliance on ability to reflect back retrospectively as a young adult on the eating disorder treatment experiences of their adolescence. This retrospective design was not relevant for parent participants, all of whom were currently supporting their autistic child/ren in paediatric services.

### Future directions

Additional qualitative research is recommended to explore the eating disorder care experiences of autistic young people who are currently in paediatric treatment, as well as the eating disorder treatment experiences of autistic young people from diverse cultural backgrounds. Participatory research that actively involves autistic young people as co-investigators throughout all aspects of the research process, is also needed to further define ongoing research priorities in relation to autism and child and adolescent eating disorders [38, 84, 85].

Interested research participants from this study along with the authors of this paper will be further developing the treatment and therapy adaptation ideas highlighted in this study through a systematic co-design process. This work will involve child and adolescent-specific implementation of the foundations of safe and supportive eating disorders treatment for autistic young people and their families, as well as the development, implementation, and evaluation of therapy modules that address important themes in the interplay of autism and eating disorders.

## Conclusion

This is the first study to focus specifically on the adolescent eating disorder treatment experiences of autistic people and parents of autistic young people. A variety of treatment experiences were explored and it was evident that autistic young people and parents of autistic young people with eating disorders face a lack of understanding across multiple domains which has ramifications for their wellbeing, their identity, their family, and their treatment. Autistic young people and their families feel supported and safe in treatment that is underpinned by treatment and clinician characteristics that promote connection and nuance, alongside autism-specific adaptions and proactive supports that strengthen that connection and provide optimal conditions for effective treatment.

### Author reflexivity statements

Andrew Wallis: I am a neurotypical white cismale and a clinical social worker and researcher in a tertiary eating disorder service. I have 25 years’ experience providing therapy for eating disorders and specialise in family therapy approaches.

Phillipa Hay: I am a clinical academic Psychiatrist with experience of caring for many people with eating disorders in general hospitals and outpatient private practice settings. I have been a lead author of Australian guidelines and other publications endorsing the need for new person centred and flexible approaches in care.

Deborah Mitchison: I am a cisfemale white clinical psychology researcher and academic specialising in the field of eating disorders. My research is particularly interested in better understanding the phenomenology and treatment of eating disorders and related issues in people from marginalised backgrounds.

## Electronic supplementary material

Below is the link to the electronic supplementary material.

Supplementary Material 1

Supplementary Material 2

## Data Availability

The datasets generated and analysed during this study are not publicly available due to the sensitive nature of the dataset.

## Footnotes

1 Throughout this research identity-first language will be used as well as the term autism instead of autism spectrum disorder. This is the preferred use of language of the autistic community and delineates a position of difference not deficit [2] Monk et al. 2022)

## Abbreviations

AN: Anorexia nervosa
ARFID: Avoidant restrictive food intake disorder
AP: Autistic person
CBT: Cognitive behaviour therapy
ED: Eating disorder
FBT: Family based treatment
GP: General practitioner
HREC: Human research ethics committee
MDT: Multi-disciplinary team
NDIS: National disability insurance scheme
OT: Occupational therapist
PEACE: Pathway for eating disorders and autism developed from clinical experience
SCHN: Sydney children’s hospitals network
