# Supplementary material for: Understanding the child and adolescent eating disorder treatment experiences of autistic people and parents
Source: J Eat Disord. 2025 Jul 6;13:128. doi: 10.1186/s40337-025-01331-w (PMC12232722; doi:10.1186/s40337-025-01331-w)
Supplement: Supplementary file 1 — Supplementary Material 1 [file 40337_2025_1331_MOESM1_ESM.docx]

**Supplementary materials - Interview outline**

**Interview questions** **– autistic person**

Are there any sensory or environmental accommodations you would like me to make for this interview?

What gender do you identify as? And what pronouns would you like me to use?

What cultural background do you identify with?

How old are you?

1. Can you please tell me about your experience of being diagnosed with autism? How has having your autism formally recognised impacted you?
2. How old were you when you developed the eating disorder?
3. How would you describe the process of getting your eating disorder diagnosis?

*What was it like being diagnosed with an eating disorder?*

*Who made the diagnosis?*

1. How have you seen your autistic traits and eating disorder interacting?

*Have your autistic traits impacted your eating disorder symptoms in anyway?*

1. Can you please tell me about your eating disorder treatment experiences?

*What eating disorder treatment/s have you experienced?*

- - What was good about it?
  - What could have been better?

1. What was your experience around your autism-related support needs*being recognised, understood, and accommodated throughout treatment?

*What is your experience of health professionals adapting their care to support your needs as an autistic person?*

1. Were there things that you wish had been considered or adapted as part of the eating disorders treatment? (in relation to autism, or gender, or other things)
2. What things did your parents/carers do that you felt were helpful and understanding of your autistic identity and support needs? Was there anything that your parents/carers did that wasn’t helpful?
3. If there was to be a treatment component developed, specifically for parents and carers of autistic children/young people with an eating disorder, what do you think it should include?
4. What other treatment components might be helpful to support autistic young people experiencing eating disorders?
5. Is there anything else you would like to reflect on?

* support needs could include: double empathy, masking, sensory, alexithymia, same foods

**Interview questions – parent**

1. Which of your family members have/had experienced an eating disorder?

*Who in your family has or has had an eating disorder?*

1. How would you describe their eating disorder (what was the diagnosis)?

*What eating disorder diagnosis do they have?*

1. At what age was the eating disorder diagnosed?

- When was this?

1. Were you able to receive any support as a parent/carer after they received their eating disorder diagnosis? Why or why not?

*(Some examples of support might be referral to a support group, inclusion in child’s eating disorders treatment, opportunity to meet with health professionals, access to own therapy)*

1. Please tell me about your child’s eating disorder treatment?
   - What did their treatment involve?
   - What was your experience of their treatment, as a carer?
2. At what age did your child receive their autism diagnosis?
3. Do you get any post-diagnostic support for yourself or your family member after their autism diagnosis?

- If yes, what support, and was it helpful?

1. How have you seen the autistic traits and eating disorder interacting?

*Has your child’s autistic traits impacted their eating disorder symptoms in anyway?*

- - How has this impacted you in your caring role?

1. What was your experience of the recognition, understanding, and accommodating of the autism-related support needs of your family member by healthcare professionals?

*What is your experience of health professionals adapting their care to support your child’s autistic needs?*

- - What was good about it?
  - What could have been better?

1. If there was to be treatment component developed, specifically for parents and carers of autistic children/young people with an eating disorder, what do you think it should include? What format would you think would be best?
2. Are you autistic? If yes:
   - What was your experience, as an autistic person, of your child’s eating disorder treatment?
   - What was your experience of the treating teams’ understanding of neurodiversity and neurodivergence?
   - What was your experience of the treating teams’ understanding of your autistic identity?
