# Supplementary material for: Understanding the child and adolescent eating disorder treatment experiences of autistic people and parents
Source: J Eat Disord. 2025 Jul 6;13:128. doi: 10.1186/s40337-025-01331-w (PMC12232722; doi:10.1186/s40337-025-01331-w)
Supplement: Supplementary file 2 — Supplementary Material 2 [file 40337_2025_1331_MOESM2_ESM.docx]

| **Major Theme** | **Theme** | **Quotes** |
| --- | --- | --- |
| Misunderstood | Problems related to autism diagnosis | “If there’d been like a bit more understanding even just from like that kind of assessment about giftedness, then maybe there would have been more exploration into like, well why does she actually think like that? And like you know, is that neurodivergence?” Lauren, AP  “No one at school ever picked up on it so I I just thought I was overreacting because no one ever picked up on her autism.” Shae, parent  “I was the one that pointed it out to the clinical psychologist.” David, parent  “I’ve suffered my whole life and I just didn’t know why.” Ari, AP  “There are a lot of autistic children and adults like myself who are being misdiagnosed and treated for things are not actually the core primary condition and therefore developing even unhealthier relationships with food when it could have been really supported at the beginning.” Gina, parent  “I’d had depression identified when I was 12 or 13 which really I think now was autistic burnout.” Bree, AP  “I don’t believe I have OCD…it’s more of the autismness of the perfect routine fix sort of thing.” Ari, AP  “Even that psychologist there was like “Oh no, Bree’s got too much social insight to be on the spectrum.” Bree, AP  “They will very much have a stereotyped view of what autism is, and I keep saying to them, you don’t understand, it’s so nuanced.” Michelle, Parent  “There was a lot of stigma.” Lauren, AP |
|  | Siloed expertise | “we need the right skills in the professionals to support us.” David, parent |
|  | Limited knowledge and treatment options for non-AN eating disorders | “The head of service essentially said, “look, we don’t treat autism. There’s no recommended treatment for ARFID. Best we can do is just offer FBT until we can hand her off to services that deal with autism.”  Michelle, parent  “So we really began to worry that it it wasn’t just you know, we could be fobbed off with fussy eating anymore. There was something seriously, psychologically going on around food and we couldn’t help her. So that was quite scary.” Wendy, parent  There are a number of eating disorders, and it would be good if we got to get funding and treatment for all of them. Restrictive might be the most common but it’s not the only one.” David, parent |
|  | Misattribution | “It’s asking them, “why do you do this?” and like I could have answered that if someone had ever asked me why, why do you use small spoons? My answer wouldn’t have been like so I have smaller bites, so I eat slowly. The answer would have been like I just like the way they feel in my mouth. I don’t want to eat with big spoons because it’s too much of a mouthful. I don’t like them. I don’t like the shape of them. I don’t like how they sit.” Frey, AP  “Sometimes doing therapy on a particularly negative behaviour can affect the autistic behaviour that shouldn’t need to be fixed. You know eating disorders need to be fixed but autism doesn’t. We can make that mistake a lot.” Alex, AP  “I found that very frustrating that they keep putting it down to autism as in like the interoceptive confusion, rather than taking the time to really think about what they’re saying and looking at the person in front of them and what is actually happening for them.”  Michelle, parent |
|  | Autism not accommodated | “Some things have been accommodated but most things haven’t just because of how little we know.” Chloe, AP  “It was acknowledged but never accommodated for. But none of the treatment I was in was autism ever like actually accommodated or understood.” Samantha, AP  “I actually vetted the dietitian before we went to ensure that she had worked with teenager girls and autistic teenage girls and ARFID, and that she knew the complexities of how they meshed. Well, the first thing that we came across the the environment was sterile, really bright lights and white, and you know really clinical, which was not good. And Elle who is extremely anxious out in society was headphones on and phone doing her game, on a game on a phone. And the dietitian told her that she had to put her phone away and look at her and take her headphones off. That was the first three things out of her mouth.” Wendy, parent  “I mean the environment I wouldn’t say was very adaptive.” Alex, AP  “I wasn’t given any fidget toys or anything.” Emerson, AP |
|  | Neuro-normative definitions of eating and recovery | “it sometimes felt like this one fits like, you’re going to fix AN. You’re going to be be good, done. That’s a blip in the road done. But I think for a lot of autistic people food is never going to be the same way, like, you know you see lots of girls who are doing these recovery journeys and they’re like learning food freedom and loving food again and it becomes very deceptive what be might achievable as an autistic person with sensory issues over food.” Alex, AP  “In the local hospital like they’ve just got I don’t what they have what they follow but it’s the same rules for everyone.” Shae, parent  “Sometimes I also think that eating disorder therapy can sometimes be a bit like you know you get it done in the 20-30 sessions but fir autistic people it could take a lot longer.” Alex, AP |
|  | One size fits all approach | “It’s not a one size fits all approach. And it just seems to be be – that’s what you’re forced into. You just feel like you don’t belong anywhere.” Rachel, parent  “When it came to health professionals at the start, I don’t think, I mean they could have been changing the way things were done but I still felt very much like it was just, I was still just me doing the same thing as everyone else.” Alex, AP  “Straight away they tried to do the FBT. IT was so head on and confrontational. She was actually shutting down; she would actually hide behind my back and she was already diagnosed as selectively mute. But they would trigger that shutdown at every single appointment. It took me a year of advocating for them to actually change the approach with FBT.”  Michelle, parent |
|  | Lack of consideration of family neurodivergence | “I think for autistic parents it’s helping them identify they’re autistic in the first place or neurodivergent. And it’s also putting it in their lap because when you are having to exhaust your own capacity managing your child’s treatment, you do not have the capacity to invest in yourself.” Michelle, parent |
|  | The impact of being misunderstood | - *Increased burden of case-management and advocacy*   “Self-advocacy skills once you are aware are really important because you’re not being difficult by asking for accommodation sometimes.” Bree, AP  “I am advocating heavily for my children. In that calorie in is the goal. And so how that looks, how they do it – if they’re sitting down, if they’re standing up, if they’re on one leg – it doesn’t matter.” Gina, parent  “I feel like so much energy is being spent advocating for the services for your child that you totally neglect what you also need as your family.” Michelle, parent  “I do feel like we had good outpatient support. But part of that is because we’ve been really pushy and proactive and bought some of that.” Selena, parent  “I’m holding all the responsibility. You’ve got to be a psychologist, a therapist, a psychiatrist, and a doctor, and you know, like it is, what other illness is like that? That you have to take so much on board?” Jackie, parent  “If people aren’t proactive in their own, finding their own knowledge, I, I don’t know that they do.” Wendy, parent  “So as a parent I’ve got no idea what to do next. I’ve got no way forward. I, the whole system is confusing – I didn’t even know the difference between a psychologist and a psychiatrist. So you’re in that place of complete disempowerment and yet you know what your autistic kid needs…And only you know that and you know it better than the nurse or the doctor or the psychiatrist. So somehow empowering parents to be advocates for their autistic child inside that cyclone of just being completely confused and shock.” Selena, parent   - *Identity disruption*   “I created myself to what environment I was in.” Alex, AP  “They [parents] don’t understand or you’re like really tired and like masking.” Emerson, AP  “The fatigue of masking or the fatigue of social processes. Yeah, that’s another thing that I wish clinicians knew that just, I have to work harder at social interactions than they do, than I think they do. And that may be why we turn to other mechanisms to try and recharge, or not feel so exhausted.” Bree, AP  “I am slowly unmasking..I’m like so I do seem more autistic but that’s because I’m allowing.” Ari, AP   - Mistrust of health professionals   “She didn’t trust them so she wouldn’t give them anything.” Jackie, parent   - Distress and trauma   “And I know that there is always distress when you’re dealing with people with eating disorders, and they’re malnourished. But the level of distress far exceeded because there was also looking back that distress was being caused by being autistic and not being accommodated and not having reasonable adjustments. It did a lot of harm.” Michelle, parent  “I don’t think people realise just how much suffering is underneath these behaviours and thoughts.” Bree, AP  “I just feel like we are at sea.” Lisa, parent  “the major sensory overload which is huge…and I’d mask that.” Ari, AP   - Set backs and reduced opportunity for recovery   “I was getting notes home every day. ‘This is not a healthy lunch’ and I’m like you don’t understand how hard I’ve worked to get him to eat this…And I was just constantly like, I’m back at square one again.” Gina, parent  “I think if I’d known that I had autism then my ability to kind of identify in myself what were meltdown and like how I could, what was a more efficient means of coping with them, like I think that would have been improved. I think because I didn’t know what was how I was feeling and why I was feeling it like I was kind of more inclined to feed into actually using eating disorder behaviours.” Lauren, AP |
| Safe and supportive eating disorders treatment for autistic young people and their families | Key foundations for treatment | - Accessibility   “Cost is a massive impact in that because often if you do get NDIS funding, they won’t cover that or you end up on a wait list forever. And just a lack of programs in general.” Gina, parent  “So when it comes to the eating disorder plan, I don’t have a paediatrician to go to. I can’t get into one. I’ve emailed quite a lot, maybe ten. I can’t get in.” Rachel, parent  “I wouldn’t say we were well off but we’re certainly privileged but we can’t afford that service. So how accessible is that?” Wendy, parent   - Listening and responding   “It took a while to get into the rhythm of that but as time went on, I felt like we were really being listened to.” Selena, parent  “It’s just listening from the start.” Jackie, parent   - Compassion   “I am really passionate about that everyone gets the same listening and compassion and connection.” Jackie, parent  “They were amazing. They’re just so understanding and supportive and understand the needs of child with what is it, neurodiversity. And I try and replicate what they say and do.” Shae, parent   - Tailored approach   “It would be nice to be told at the start that ‘there’s more than one way to do this. And that it’s okay you know, it’s okay to choose your path.” Rachel, parent  “It would have been much better if that was a much more individualised approach.” Frey, AP  “The autism diagnosis should have informed and empowered. It should have triggered an immediate review of care to become more individualized, responsive to complexities, and mindful of nuance.”  Michelle, parent  “Not all of us are the same. One person with autism is one person with autism.” Gina, parent   - Holistic care   “I’ve gone through all the different types of treatment and CBT and introducing foods and all of that but nothing worked because…I don’t think they were treating what they were meant to be treating. It’s not their fault but yeah, they were just treating an eating disorder without the autism factor.” Ari, AP  “Look at it holistically like not just looking at the eating disorder but like, and not just looking at autism.” Samantha, AP  “The clinician we work with now she’s pretty good but she she’s made it clear that she’s there to help with the eating disorder. When we bring up outside things that are impacting her, she’ll say, she’ll just say “make, like try and follow up with your NDIS application to get help with that.” Shae, parent   - Humility   He [doctor] wasn’t good. Like you know when people talk to you something they talk in a way that’s like ‘I’m better than you. I’m this, I’m that.’ And he just very much had that quality.” Clara, AP  “There was a lack of humility in how she approached Maisie’s eating…I felt like she probably would have been more helpful had she come in being curious to learning about how this is for Maisie and then applying her knowledge versus applying her knowledge without taking the time to identify why Maisie’s safe foods were safe and why some foods are unsafe.” Michelle, parent |
|  | Autism-specific foundations for treatment | - Neuro-affirming care   “Those psychs also just operating just in like neuro-affirming frameworks.” Frey, AP  “In the concept of learning how to unmask is that sometimes, or for me specifically, especially is giving me permission that I’m allowed to have a choice.” Alex, AP  “Communicate that they may not want to make eye contact. Yeah, they don’t have to because that a real big, neuronormative thing about being polite.” Bree, AP   - Accommodations   “If there are just some adjustments, some little things that we can do that make a big difference.” Selena, parent  “For me at least the things that I needed were adapted.” Clara, AP   - Relevant information and resources   “There’s so much stuff out there though it’s like, you know, managing what do I look at? And what don’t I? Because you can nearly be consumed by it.” Jackie, parent  “There isn’t one point of contact. There’s information splatter.” Wendy, parent  “Neurodiversity affirming information about what autism is.” Bree, AP  “I remember I was trying to find out more about autism and Eds so I actually went on TikTok and I got pulled down a really terrible rabbit hole.” Emerson, AP  “I think it’s useful to have like information resource booklets. I think if they were something that was kind of like had the information, and had suggestions and reasons why things were like accommodations…Like things that aren’t just information resources. They’re very much like here is all the information you need to be able to determine this for yourself.” Frey, AP   - Case management and navigation   “Partnerships are really important in schools between people who work with certain kids. Yeah, once autism is identified I think the school should be informed. I guess within a neurodiversity affirming lens too to support that child.” Bree, AP  “The NDIS response is often not very helpful as well, and they’ll often put it back to parental responsibility.” Gina, parent  “Help with NDIS. God help us!” Wendy, parent   - Working understanding of autism and eating disorders   “Holistic clinicians that can treat both.” Shae, parent  “More advice around binge eating disorder would be good and some advice around how it interacts with autism.” Lisa, parent  “The local eating disorder coordinator, I believe she actually ran like a seminar on how to actually work…with neurodivergence in general but she pretty much went in and educated like all of the psych ward staff on effectively how to like respond to me…I didn’t really have any negative experiences when the nurses that that was something that was kind of out of my control.” Lauren, AP |
|  | Informed by lived experience and opportunities to connect with other autistic people | “Opportunities to have discussions and to meet people who have similar experiences because a lot of us feel incredibly isolated and alienated and othered by the world around us. So I feel like getting that connection is huge.” Frey, AP  “You can feel a bit alone and part of like an eating disorder and just mental illness in general is kind of like isolating yourself and so to have more opportunities to meet other people in like safe spaces or just like feel understood because I think for the most part that’s lacking in a lot of spaces.” Emerson, AP  “Maybe if someone that’s autistic adult now can share their experience.” Ari, AP |
|  | Therapy considerations | “I think as well it would have been nice to have a bit more like in a way like understanding about special interests and learning into those…and you might not feel like you can explore or talk about them with like friends or family…and so part of support and like counselling because like to create space were you can engage with it a little bit.” Emerson, AP  “And maybe having a structure and a predictability. So this is what we’re working on in the treatment so that they can look ahead, or they know what the agenda is rather than just, oh, we show up, and then we talk about whatever comes up.” Bree, AP  “giving them the skills and the confidence to be able to advocate for their own needs.” Frey  “Not just treating the person with the diagnosis but also treating their support network. You know, with information and training and strategies and ideas of how they can help. It has to be a family-based approach.” David, parent  “I do think it’s very important to have like parents involved and like understand because when you’re young like you were in the home and they it’s something that’s like deeply impactful on your life, but I think as well kind of like having some forms of control over that.” Emerson, AP  “It was obviously helpful to have my parents engage in the therapy at times because I think it also taught them a lot about themselves. And I think it did help them relate better and be able to assist better when you can recognize your own things in yourself.” Alex, AP  “There should be some sort of solo therapy with a therapist and the person alone. Like if you’re going to have to have family therapy there should be a solo therapy as well. I think as well there should be a separate thing for the families to actually understand the not just eating disorders and not just autism but both of them and how they come together because I think there’s not enough understanding.” Samantha, AP  “I hadn’t really worked out the roots of why I developed the eating disorder and like how to manage it. I quickly spiraled.” Chloe, AP  “I didn’t see my eating disorder as separate part to me as in a different person. And so it felt like I was being denied my own voice because they kept labelling it as a separate voice and I never understood that. I know it’s helpful for some people to give it a separate but for me it felt like me being shut up.” Alex, AP  “I think a really really life changing thing is externalising the eating disorder.” Bree, AP  “I feel like even like group like talking circles could even be helpful. I know my mum felt a bit alone I think ‘cause it’s kind of difficult to talk to other parents and…. she didn’t even know like how to get me help.” Emerson, AP  “I think it’s important for parents to kind of like have an understanding of you know the ways that distress manifests.” Lauren, AP  “Some sort of education on both autism, eating disorder separately and then together. Because at least understanding how your child’s brain is working or how your sibling’s brain is working to begin with, and then how you know how eating disorders work. For the family to understand that would probably really help in being supportive of the person with the eating disorder.” Samantha, AP  “Understanding like the control is what’s needed. And so how can we give you control in other ways because I feel like a lot of the treatments kind of further that out of control feeling.” Emerson, AP  “How can we use those traits [autistic traits] to help her and actually not, not treat her like she’s a naughty little girl.” Rachel, parent  “You can sort of like appeal to your autistic traits in the person or work with those traits and that’s going to look different from every person.” Samantha, AP    “I like how therapy isn’t just like one thing. Like we do art and stuff as well.” Clara, AP  “They would say, ‘Let’s get down to the root cause of this. Like the one that said blame on the sister’ because she’s you know prettier than me…and then it would be about my peers, or what I see on TV. And I’m like, no, I don’t, it’s got nothing to do with that…looking at other reasons why people have eating disorders, I guess. And a big thing of power is control. I couldn’t control my life because I didn’t really know who I was or what I am but that was the one thing I could.” Ari, AP |
